# Vulnerability assessment of agricultural production systems to drought stresses using robustness measures

**DOI:** 10.1038/s41598-021-98829-5

**Published:** 2021-11-04

**Authors:** Marangely Gonzalez Cruz, E. Annette Hernandez, Venkatesh Uddameri

**Affiliations:** Department of Civil, Environmental and Construction Engineering, Texas Tech University, Lubbock, TX 79409-1023 USA

**Keywords:** Natural hazards, Environmental impact, Climate-change impacts, Hydrology, Civil engineering

## Abstract

Intensification of droughts in agricultural areas threaten global food security. The impacts of drought stresses vary widely across a region, not only due to climate variability but also due to heterogeneous soil and groundwater buffering capacities which protect against droughts. An innovative drought vulnerability index was developed by reconciling the negative effects of drought stresses against the robustness offered by hydrologic buffers. Indicators for climate stresses, soil and groundwater buffering capacities were defined using physical principles and integrated using a multi-criteria decision making (MCDM) framework. The framework was applied to delineate drought vulnerability of agricultural production systems and evaluate current cropping choices across the High Plains region of the US that is underlain by the Ogallala Aquifer. Current crop growth choices appeared to be compatible with the intrinsic drought vulnerabilities with cotton and sorghum grown in higher vulnerability areas and corn and soybean produced in areas with lower vulnerability. Nearly 50% of the aquifer region fell in the transition zone exhibiting medium to high vulnerabilities warranting the need for better water management to adapt to a changing climate.

## Introduction

Sustaining and improving agricultural production is critical to continue meeting the ever-increasing food, fiber and fuel demands of the planet. Climate change is now recognized as a major threat to hydrological and agricultural production systems (APS) around the world^1–5^. While the impacts of climate change are multifaceted, the increased frequency and intensity of droughts that are being projected in many parts of the world, represent a major threat to APS, especially in arid and semi-arid regions^6–9^. Moisture deficits caused by droughts and climate induced shifts in rainfall patterns critically affect the amount of water that is available to plants, especially during critical growth stages^10,11^. In addition, increases to atmospheric temperature and solar radiation further aggravate crop water needs and affect the performance of APS.

Plants have certain intrinsic coping mechanisms both at the cellular and organism levels to handle water and heat stresses caused by droughts^12^. However, plant growth, in general, is highly sensitive to water deficits, primarily due to cell elongation^13^. Prolonged droughts usually affect plants in many different ways including but not limited to reduced leaf area, stunted canopy and root development, as well as diminishing the amount and quality of fiber^14^. Droughts are noted to reduce both the harvested area and yields. Studies have shown that droughts have reduced cereal yield by roughly 10%, globally^15^. Recent droughts have led to economic losses in the agricultural sector that are estimated to be in the billions of dollars^16,17^. Understanding the vulnerability of APSs to droughts is important to sustain and improve global food production.

The vulnerability of APS to climate stresses is defined as their inability to withstand droughts, in this study. This definition of vulnerability is related to the robustness of the system. Robustness is commonly defined in engineering literature as the ability of the system to maintain its performance when subjected to external disturbances^18^. An APS is said to be robust if the crop yields are not significantly affected by droughts; otherwise, it exhibits vulnerability. Vulnerability has been used as being opposite to robustness in water related studies ranging from climate to agriculture^19–21^. Therefore, robustness measures provide an excellent platform to study the vulnerability of APS to droughts.

The primary factors that build robustness in APS are water that is stored in the root zone of the soil at the initiation of the drought and external water supplies from storage that can be used for irrigation. While both surface and groundwater resources are used for irrigation, this study focuses on groundwater alone. Groundwater is heavily relied upon to meet agricultural water demands across the world and is often the only source of water in many arid and semi-arid regions of the world^22–24^. While droughts do impact aquifer water levels^25^ groundwater is often viewed as a buffer resource as the intrinsic response of aquifers to droughts is slower^26^ and therefore is relied heavily to meet increased water demands. Therefore, groundwater extraction is known to increase substantially during periods of droughts^27,28^.

Both soil moisture storage and groundwater reserves can be heterogeneous within a region. Therefore, different APSs, within the same region, exhibit varying levels of robustness to withstand a drought of a given intensity. Clearly, the severity and magnitude of droughts also vary across a given area of interest. Therefore, the vulnerability of an APS can be viewed as a net effect of drought stresses and hydrologic buffering capacity which is influenced by soil characteristics and groundwater availability within a region. The study uses physical principles to identify droughts (stresses) as well as soil and groundwater (robustness) indicators which are then integrated using a multi-criteria decision making (MCDM) framework to properly assess the competing goals of climate stresses and hydrologic buffers using the developed vulnerability index (VI).

The developed methodology is presented in detail and illustrated by applying it to map drought vulnerability of the Ogallala Aquifer. Ogallala Aquifer is the largest aquifer in the United States that not only sustains the rural economy over eight states but also provides food, fiber and fuel supplies across the world^29^. Therefore, the study not only develops a new theoretical framework for assessing robustness of APSs, but also illustrates its utility by applying it to a major, groundwater-dependent, food producing area in the world.

## Methodology

### Robustness-based Vulnerability Index

**Figure 1:**
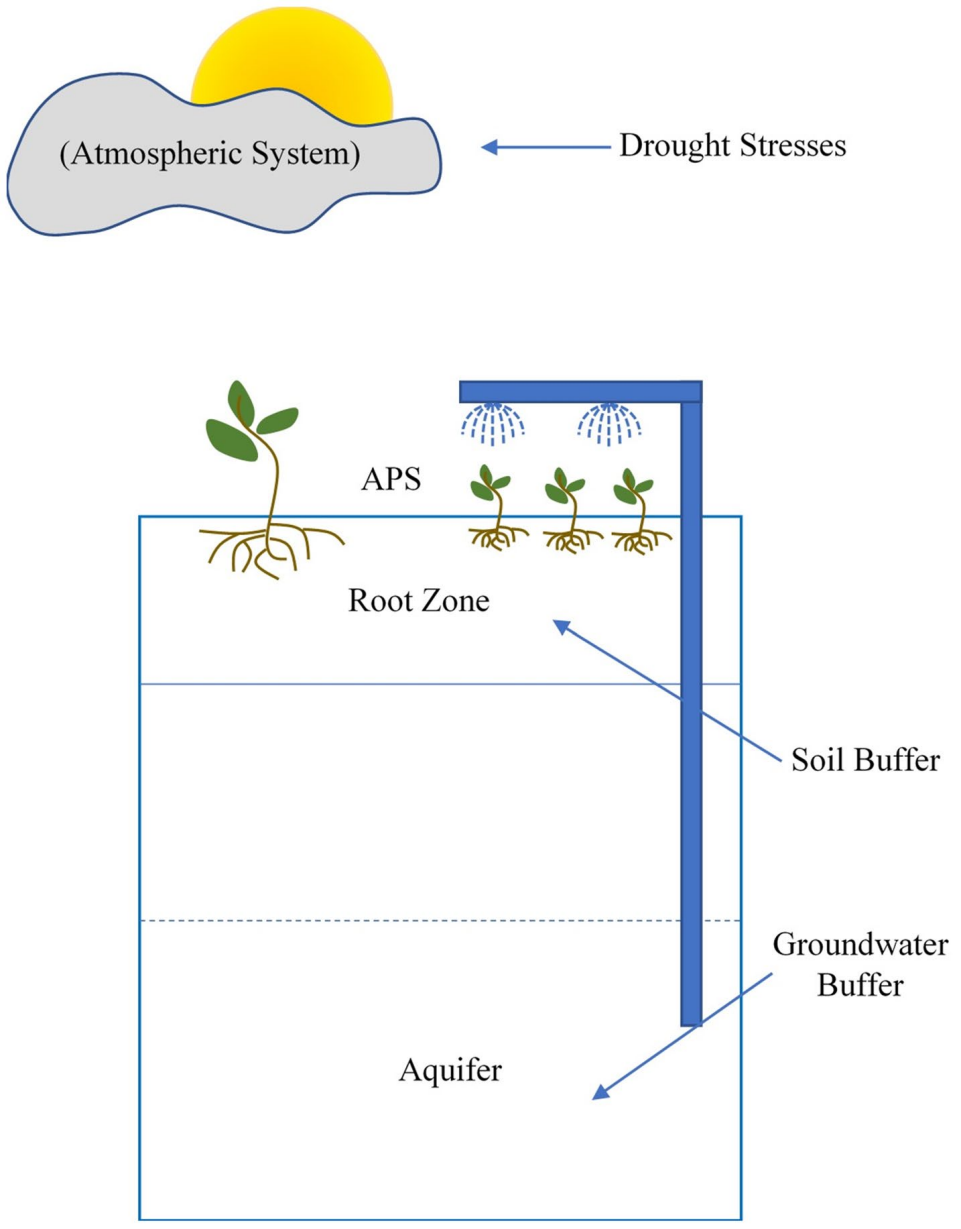
Conceptual model of the agricultural production system (APS) and hydrologic buffers.

A conceptual model of the APS system of systems is depicted in Fig. 1. The climate system induces water stresses through droughts. The water available in the root zone at the initiation of the drought provides the first line of defense against the induced climate stress. If the moisture is above the permanent wilting point (PWP), plants can readily access this water to meet the increased water demands brought forth by droughts. However, the buffering capacity of the soil is finite, and the available moisture in the root zone can be depleted due to evapotranspiration (ET). In such instances, additional water from an external source (e.g., groundwater) must be added to ensure sufficient water is available for plants to meet their water demands. It is assumed that a farmer will meet this irrigation requirement using groundwater supplies available at the farm in this study as it often represents the most reliable source of water.

To capture the net competing effects of climate stress and hydrologic buffering capacity, Vulnerability Index (VI) is defined here as the ratio of the Drought (climate) stress to the weighted sum of the buffering capacity offered by soil and groundwater sources:

1 $$VI= \frac{DSI}{\left(\alpha SBI+ \beta GBI\right)}= \frac{1}{RI}$$

where VI is the vulnerability index, RI is the robustness index, DSI is the drought stress index, SBI is the soil buffer index and GBI is the groundwater buffer index. DSI captures the water deficit caused by the drought stress while SBI and GBI capture the effects of soil and groundwater buffering capacities, respectively, at a location of interest. It is also important to note that both SBI and GBI are to be evaluated at a specified point of time (e.g., a drought event of interest) to ensure their consistency.

The variables α and β vary between 0 and 1 and weigh the relative contributions of soil and groundwater buffering indices such that α + β = 1. If the numerator and denominator terms of Eq. (1) are measured on the same scale, then values of VI greater than unity indicate a vulnerability to drought stresses and those below unity the robustness of the APS. For regional-scale comparisons, DSI, SBI, GBI can all be normalized to vary between 0 and 1 to facilitate consistent spatial comparisons. The reciprocal of VI is termed the robustness index, RI.

Robustness is defined here as the ability of the APS to withstand external drought stresses. This definition of robustness has also been used to describe the resilience of a system^30^. Robustness is preferred over resilience in this study because it has been defined more consistently in the literature and has a clearer linkage to sensitivity, or the response of a system to external stresses^31^. The buffering capacity to withstand external stresses (i.e., robustness) can be viewed as one of the many dimensions of resilience^32^. The vulnerability index (VI) represents conditions at a given point in time and corresponds to static vulnerability^33^. The computation of VI requires specification of indices for Drought stresses and hydrologic buffers which are discussed next.

### Drought Stress Index (DSI)

The numerator of the proposed vulnerability index (VI) captures the Drought stresses of the aquifer. Meteorological droughts are commonly understood to be moisture anomalies of below average precipitation and/or above normal temperatures over a sufficiently long period of time^34–36^. Many indicators have been proposed in the literature to characterize droughts, and a single indicator is often insufficient to fully capture the complex nature of the drought phenomena^37^. Furthermore, as droughts manifest at multiple time scales, it is also important to use drought indicators computed over different moisture accumulation periods. For example, 3-month and 6-month accumulations of Standardized Precipitation Index (SPI)^38^ and the Standardized Precipitation Evapotranspiration Index (SPEI)^39^ are commonly used to calculate intra-season and full season agricultural droughts^40,41^.

The severity of the drought (DS) and the duration of the drought (DD) are two fundamental characteristics of droughts^42^ as they capture the intensity of a drought event. The inter-drought duration (IDD) can be defined as the time between two drought events, i.e., time between the cessation of a drought and initiation of the next. The duration and severity of droughts are not constant at a given site. Therefore, representative metrics are necessary to capture the long-term behavior and make consistent spatial comparisons. The first statistical moment or expected value (a probability weighted mean) is a widely used metric in decision analytic theory to compare the outcomes arising from random variables^43^. It has been used to summarize climate related information in agricultural decision making^44^ and as such adopted here, as well.

The expected values of the drought characteristics can be computed using Eq. (2):

2 $$E\left({c}_{i,j,k}\right)= {\int }_{0}^{\infty }{c}_{i,j,k} f\left({c}_{i,j,k}\right)$$

where E(c_i_) is the expected value of the ith drought characteristic (i = DS, DD, IDD) and subscripts j,k are indices for the drought indicator (j = SPI, SPEI) and the accumulation period of the drought characteristic (k = 3 Months and 6 Months), respectively.

While variations between different meteorological drought indicators are to be expected due to differences in their mathematical constructs, correlations between them are also very likely as these indicators often share similar datasets and are all trying to characterize moisture deficits in the atmosphere. As such, the Drought stress index, DSI, at location, *l*, is calculated as the weighted product of the expected values of drought characteristics for adopted indicators (Eq. 3).

3 $${DSI}_{l}= \prod {E\left({c}_{i,j,k}\right)}^{{w}_{i,j,k}}$$

where w_i,j,k_ is the weight of the ith characteristic (i = DS,DD,IDD) of the jth indicator (j = SPI, SPEI) and kth accumulation period (k = 3-month, 6-month) computed at location, *l*. The weights of severity and duration need to be positive (higher values imply higher climate stresses) while that of the inter-drought duration needs to be negative because closely spaced droughts are difficult to cope with and therefore add greater stress to the APS. The use of the product weighted aggregation (Eq. 3) is also beneficial in that it does not require the attributes to be normalized prior to their weighted multiplication.

### Soil Buffering Index (SBI)

The soil buffering index represents the relative buffering capacity of the soil against climate-induced stresses across a domain of interest. The soil buffering index is developed here using the ideas of stocks and flows as they are basic building blocks for studying system dynamics^45^. The ability of the soil to thwart climate stresses clearly depends upon how much water is potentially available in the root zone (a stock indicator) and, also, how the soil moisture likely responds to climate stresses (rate or flow indicator).

The amount of water that can be held in the root zone represents a useful measure to compare the buffering capacity between two different soils assuming all other factors being the same^46^. The plant available water (PAW) in the root zone is the amount of water that is held between field capacity (gravity drainage threshold) and permanent wilting point (root uptake threshold) and is, therefore, calculated as PAW = (field capacity) − (permanent wilting point). PAW is taken here to represent the stock component of the soil buffering capacity. PAW measures the intrinsic ability the soil to hold water and higher the PAW the greater is the potential likelihood of the soil to withstand the drought, *(all other things being equal)*. PAW values are often reported in units of length (e.g., mm) in soil science literature, it is important that such dimensional PAW values be divided by the depth of the root zone to make a consistent comparison across different locations as root depths often vary widely within a region.

The propagation of meteorological drought through the soil profile is often referred to as the agricultural drought^47^. The soil moisture is the key variable used to define agricultural droughts^48^. Agricultural droughts can be viewed as deviations from long-term soil moisture averages for a given period of time. These soil moisture departures can be standardized to facilitate comparisons in space and time^49^.

Soils typically act as low pass filters and often react more slowly than meteorological droughts^50^. Therefore, soil moisture can both absorb the effects of meteorological droughts and continue to persist in a drier than normal state even after the cessation of a meteorological droughts. The fraction of time when there is no agricultural drought given meteorological droughts is referred to as “Drought Absorption Capacity” (DAC). The drought absorption capacity is a measure of the buffering capacity of the soil against meteorological droughts. DAC is particularly relevant when meteorological initially sets in but the soil has sufficient moisture to continue providing water to plants despite the initiation of meteorological drought.

The fraction of time when there is agricultural drought but there is no meteorological drought is called “Agricultural Drought Persistence” (ADP). ADP is particularly relevant at the cessation of meteorological droughts. The conditions in the atmosphere have changed due to precipitation and/or lowering of temperatures. However, these conditions are not sufficient to bring soils back from their dried state and the soil is unable to meet the plant water demands as it normally would. Thus, agricultural droughts continue to persist despite a respite in meteorological droughts, thus leaving the APS vulnerable to water stresses.

DAC and ADP represent the two rate indicators to capture the response of agricultural droughts in comparison to meteorological droughts and represent the long-term temporal behavior of the soil buffering capacity.

Soils that have higher values of DAC and lower values of ADP have greater soil buffering capacity. DAC and ADP values can be computed by first converting meteorological and agricultural drought time-series into binary (drought, no-drought) time series using suitable cut-offs and then developing contingency tables between them^41^. Again, correlations between PAW, DAC and ADP are to be expected as they all are based on soil characteristics. Therefore, the soil buffering index (SBI) at location, *i*, can be represented using Eq. (4):

4 $${SBI}_{l}= \prod {{PAW}_{l}}^{{w}_{l}}{\left({DAC}_{i-j,k}\right)}^{{w}_{i-j,k}}{\left({ADP}_{i-j,k}\right)}^{-{w}_{i-j,k}}$$

where the subscript, i-j,k corresponds to drought state comparison between ith agricultural drought indicator and jth meteorological drought indicator for the kth accumulation period. The weights of PAW and DAC are positive as higher values correspond to better buffering capacity of the soil while the weight of the ADP is negative because higher values indicate lower buffering capacity at the location.

### Groundwater Buffer Index (GBI)

The system dynamics concepts of stocks and flows were again used to define indicators of hydrologic buffers. The aquifer transmissivity, T is the product of saturated thickness (the amount of water in the aquifer) and the hydraulic conductivity (K) the rate of movement of this water to a well under unit gradient (Eq. 5). Transmissivity provides a physically-based estimate of water available to a farmer from a well^51^.

5 $$T=K*ST$$

Higher values of transmissivity indicate higher potential for water to flow towards a well and therefore higher buffering capacity (all other things being equal).

The extraction of groundwater causes a drop in the water level at the aquifer. Vertical drop of the water increases the energy requirements for irrigation. Furthermore, groundwater policies may also dictate that the farmer withdraws water without impacting the neighbor’s ability to do so^52^. The storage coefficient (or specific yield in an unconfined aquifer) is a measure of the volume of water that is released per unit surface area per unit drop in head^51^. The storage coefficient (specific yield) can be used to evaluate the drop in the groundwater level per unit of water that is extracted. In agricultural literature, irrigation (IRRAMT) is often measured in units of length by dividing the volume of water (V) applied over the area of the field (A). Therefore, the drop in the groundwater level per unit amount of irrigation water, IRRAMT, (measured in inches or mm) can be expressed using Eq. (6):

6 $$\Delta H= \frac{V}{SA}= \frac{IRRAMT}{S}$$

where DH is the drop in the groundwater level, S is the storage coefficient (taken to be equal to specific yield in unconfined aquifers). Smaller values of storage coefficient cause larger drops in groundwater level for a given amount of irrigation (see Eq. 6). Higher values of groundwater level drops per unit irrigation amount not only increase the energy (costs) of pumping water for irrigation but also has the potential to affect the ability of the farmer to irrigate. Significant vertical drops of water level restrict a farmer from being able to supply additional water for irrigation, thereby limiting the groundwater buffering capacity.

As both T and S are functions of aquifer hydrogeological conditions and are often simultaneously estimated using aquifer pumping tests, a weighted multiplicative model is prescribed to quantify the intrinsic groundwater buffering capacity and is expressed as:

7 $${GBI}_{l}= {\left(T\right)}^{{w}_{T,l}} {\left(S\right)}^{{-w}_{S,l}}$$

The GBI term defined in Eq. (7) reduces to hydraulic diffusivity (which is defined as the ratio of transmissivity to storage coefficient) if both the weights are assumed to be equal to unity. Hydraulic diffusivity is a measure of how fast a pressure pulse propagates through the system^51^. Therefore, Eq. (7) defines the ability of the underlying aquifer to supply groundwater considering both storage and flow and is used as a physically-based measure of its buffering capacity.

### Determination of weights

The multi-criteria decision making (MCDM) approach adopted in Eqs. (1)–(7) require specification of weights for different attributes^53^. A variety of different approaches have been suggested in the literature to obtain weights^53^. While any weighting method can be used with the proposed methodology, it is important to remember that the choice of the weighting method will have an impact on the outcomes. To avoid subjectivity, it is recommended that weights be derived objectively from the data. The entropy method^54^ is one such objective method that uses the probability distribution of the attribute to obtain weights. This method is based on the idea that normalized attributes that have sharply peaked distributions contain more information (less uncertainty) than broadly peaked distributions and as such are assigned higher weights.

To obtain weights using the entropy method, the raw (dimensional) ratings for various attributes are normalized between 0 and 1 such that 0 represents the least preferred and 1 corresponds to the most preferred alternative. In regional-scale studies normalization can be carried out using the minimum and maximum values for each index that is observed within the domain of interest.

The probabilities of each alternative for each attribute is computed using Eq. (8):

8 $${p}_{i,j}= \frac{{X}_{n,i,j}}{\sum_{j=1}^{J}{X}_{n,i,j}} where i=1,\dots ,I \left(attributes\right)\,and\,j=1,\dots , J (alternatives)$$

The entropy of the ith alternative (E_i_) can then be computed using Eq. (9):

9 $${E}_{i}= \frac{-\sum_{j=1}^{J}{p}_{i,j}\mathrm{log}({p}_{i,j})}{\mathrm{log}(J)} \forall i=1,\dots , I$$

Finally, the weights can be computed using Eq. (10):

10 $${w}_{i}= \frac{\left(1- {E}_{i}\right)}{\sum_{k=1}^{I}\left(1- {E}_{k}\right)}$$

where w_i_ is the entropy-based weight of the ith attribute. Note that the robustness evaluation framework requires weights to be computed separately for the drought stress index (Eq. 3), soil buffer index (Eq. 4) and the groundwater buffer index (Eq. 7). In addition, a separate set of weights (a and b) are needed to compute the vulnerability index (Eq. 1). Equations (1)–(10) provide the complete set of equations required to quantify the relative vulnerability of various APSs within a region to drought stresses.

### Model assumptions and limitations

The developed model (Eqs. 1–10) evaluates the relative impacts of climate stresses (caused by droughts) against the robustness (to droughts) provided by hydrologic buffers at a location. The model development sought to balance the physical basis for quantifying the effects of stresses and buffers against practical considerations of accomplishing such a comparison with available data. As such, the ratio presented in Eq. (1) is not dimensionally consistent. However, it can still be applied at a single site either using subjective weights or assigning equal weights following the Laplace principle of insufficient reason. Qualitatively, smaller values of VI indicate lesser vulnerability and larger values greater vulnerability to droughts.

The primary benefit of the proposed approach lies in making comparative (regional scale) assessments of relative drought stresses and buffering capacities across multiple APS. Normalization of drought stresses, soil buffering and groundwater buffering indices allows for computation of objective weights. Such normalization is specific to the area being considered and values must only be interpreted in a relative mode and not in an absolute sense. The relative mode comparison is common to all vulnerability indicators that utilize MCDM approaches^55^.

The model is developed using established principles and concepts drawn from climate science, soil physics and groundwater hydrology. However, the underlying basis of the model is still empirical in nature as it uses multi-criteria decision making (MCDM) approach to tie these concepts together to create a unified index. Therefore, a rigorous validation of the model (i.e., comparison of computed index with observed values) is not possible, as is often the case with MCDM methods. Therefore, the model should only be used in a relative mode (i.e., comparing values at different instances of time at a single APS or for comparing multiple APSs within a region at a single time). The model provides a single indicator to assess the relative changes of the competing effects of climate stresses and hydrologic buffering in time or space. Generally speaking, the validity of the index hinges on the reliability of the data and methods used to obtain drought indicators and using field observed and validated datasets for required soil and aquifer characteristics.

While outside the scope of this study, a formal evaluation of how uncertainties in input parameters propagate and affect the developed approach can be used to obtain uncertainty bounds of the vulnerability index^56^. The addition of such uncertainty bounds will further improve the value of the proposed index and overcome some of the limitations associated with lack of formal verification of the proposed index.

The model development here uses concepts from stochastic theory (expected values, probabilities, entropy). However, the approach is deterministic—random behavior is captured using long-term probabilities or expected values that are then treated deterministically. The reasonableness of such an approach hinges on availability of long-term datasets (at least 30 years or more) to ensure representativeness of the values being computed^38^. Finally, it is important to remember that the proposed approach considers static vulnerability (i.e., vulnerability at a given point in time at a given location).

The vulnerability index must be developed using verified, long-term data and standard methods for assessing droughts to ensure its applicability and utility. The assumptions discussed above must be borne in mind to properly interpret the results obtained from the model.

### Illustrative case study

**Figure 2:**
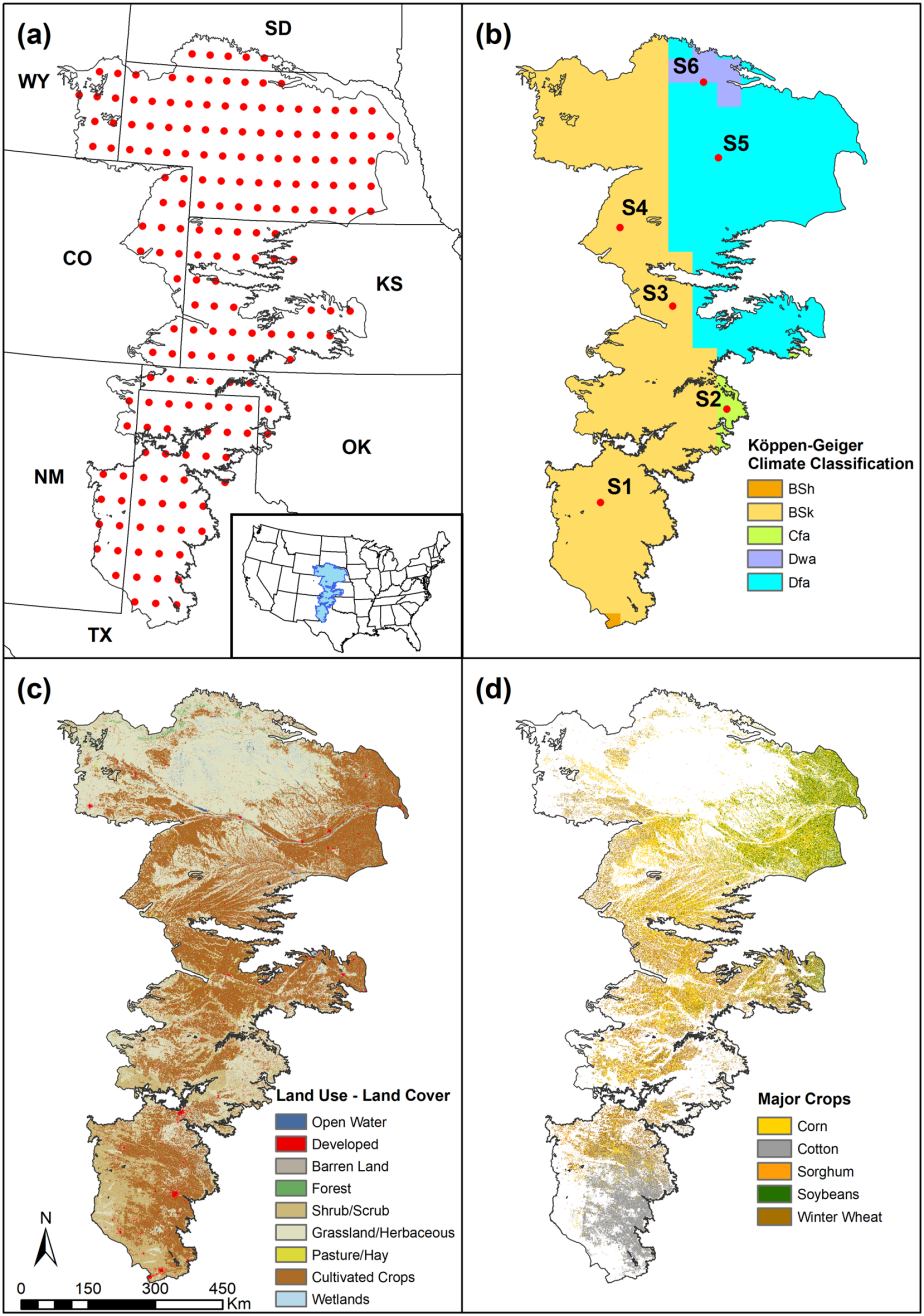
High Plains Aquifer (HPA)—(**a**) Location and Grid Points used for Analysis; (**b**) Climate Zones within the Study Area and Representative Grid Locations; (**c**) General Land Use Land Cover (LULC) ( Source: NLCD, 2016 ); (**d**) Locations within the Aquifer where Major Crops are Produced (Source: USDA-NASS Cropscape, 2019).

The High Plains Aquifer (HPA) also referred to as the Ogallala Aquifer (OA) is the largest aquifer in the United States and spans over 450,000 sq. kilometers across eight states (see Fig. 2a). The region is predominantly semi-arid (Fig. 2b) with limited surface water resources and there is a heavy reliance on groundwater from the aquifer^57,58^. Over 35% of the area is used for agriculture (Fig. 2c) and the region is a major producer of cotton, corn, sorghum, soybeans and wheat^59^ (Fig. 2d).

Overexploitation of groundwater has led to severe declines in the water table in many parts of the aquifer^57^. Recharge to the aquifer is limited due to deep water tables and limited rainfall^58^. The climate in the region is highly erratic and punctuated with frequent droughts, which have resulted in multi-billion dollar losses in recent times^16^. Predictions from global climate models (GCMs) indicate an increased dryness over much of the region^6^. Given the variability in soil, climate and groundwater availability, the vulnerability of the region to droughts and the robustness offered by soil and groundwater sources can vary widely across the aquifer.

Understanding the aquifer-wide vulnerability (or lack of robustness) is important for evaluating the viability of current agricultural practices and to develop policies and guidelines for future management of the aquifer in light of climate change. Sustaining Ogallala Aquifer is not only necessary for the vitality of this predominantly rural region, but agricultural production from this area is important for food, fiber and fuel security across the globe^29^. Extending the useful life of the Ogallala Aquifer is one of the greatest water resources management challenges faced by the United States^60^. The vulnerability mapping based on an assessment of drought stresses and hydrologic buffers is a fundamental step towards climate informed, resource appraised, land and water resources management. The Ogallala Aquifer provides an ideal test bed for illustrating the methodology developed in this study.

### Data compilation

Data necessary for computing the vulnerability index were compiled from a variety of sources (see Table S1 in the Supplementary Materials for additional details). Both SPI and SPEI computed at three and six month accumulations were chosen as meteorological indicators as they have been shown to capture intra- and full-season droughts within the region^6,40^. While SPI and SPEI exhibit strong correlation, they also exhibit differences in duration and severity characteristics and as such retained here. Furthermore, comparison of SPI and SPEI helps evaluate whether droughts are precipitation-controlled or temperature-controlled^61^. The climate indicators were computed using gridded data from Climate Research Unit (CRU version 4.03). The CRU data has been used in previous drought studies in the region^6,40^ and preliminary comparisons at select locations within the study area further affirmed its accuracy and reasonableness for quantifying droughts. The potential evapotranspiration (PET) data from CRU is based on the modified Penman-Montieth equation and therefore accounts for both solar radiation and wind effects^62^. All computations were carried out for hydrologic years 1949–2018 on a 0.5° × 0.5° grid which resulted in 187 locations across the study area (Fig. 2a).

Climate states were classified as “droughts” when SPI or SPEI values were less than or equal to -1 or as “no-droughts,” otherwise^42^. The theory of runs was used to ascertain different climate events^63^. The first and the last climate event in the record were discarded to remove partial sampling effects. The drought duration (DD), drought severity (DS) and the inter-drought duration (IDD) were computed separately at each site for each indicator at 3- and 6-month accumulations. Probability distributions were fit separately for each DD, DS and IDD datasets. Based on Akaike Information Criterion (AIC), the exponential distribution was noted to provide best fits for DD and IDD datasets and the lognormal distribution was noted to be best for DS at a majority (~ 83%) of locations along with Weibull (~ 9%) and Gamma (~ 8%) at some locations. These distributions were then used to compute the expected values for the three drought characteristics for each indicator.

The plant available water (PAW) in the root zone was computed using high resolution gridded SSURGO soil data^64^ as PAW = (field capacity) − (permanent wilting point). The normalized PAW (NPAW) was obtained by dividing the available water in the root zone (in mm) by the root zone depth^64^ (converted to mm). The standardized soil moisture index (SSMI) was computed in a manner analogous to SPI. The soil moisture data for hydrologic years 1949–2018 were obtained from the gridded soil moisture data provided by the climate prediction center^65^. The monthly soil moisture estimates are provided on a 0.5° × 0.5° grid and are consistent with the spatial resolution of the CRU data used to compute climate indices. The soil moisture predictions used for SSMI computations were based on the outputs from a leaky single bucket soil water balance model^66^. The root zone depth varied across the study area; however, the leaky bucket model assumed a typical depth of 160 cm. The model has been calibrated to watersheds in Oklahoma and as such representative of conditions within the study area. Despite its simplicity, the model is shown to predict long-term trends and variations reasonably well at many locations, including the study area of interest here^65^. This soil moisture data has also been used in previous studies^67^, to construct a standardized soil moisture index similar to the one in this study. SSMI values were calculated at 187 locations at 3- and 6-month accumulations and converted to binary time-series using a cut-off value of -1 for consistency with meteorological indicators. Pairwise SSMI and SPI/SPEI data were used to construct contingency tables and compute drought absorption capacity (DAC) and agricultural drought persistence (ADP) metrics at each location.

All groundwater data were obtained from the United States Geological Survey (USGS)^57,68–70^ (see Table S1 in the supplementary information for additional details). The depth to groundwater in the year 2013 and the changes to water table between 2013 and 2015 were used to construct the depth to water table for the year 2015 (the most recent data that were available when the study was performed). The saturated thickness, depth to groundwater were both available for the year 2009 and were used to delineate the bottom of the aquifer. The aquifer bottom in conjunction with the depth to groundwater in the year 2015 along with land surface elevation were used to obtain saturated thickness in the year 2015. Inverse distance weighting (IDW) was used to create surfaces from groundwater level data and saturated thickness information. Negligible errors (maximum error < 1 × 10^–9^) were noted between the saturated thickness surface for the year 2009 constructed in this study and the spatial data provided by the USGS indicating the suitability of the adopted method to create requisite water level and saturated thickness surfaces. The transmissivity across the aquifer was computed by multiplying the estimated saturated thickness for the year 2015 with the corresponding horizontal hydraulic conductivity. The drop in the groundwater level per 1 inch (2.54 cm) of irrigation amount was computed from specific yield data.

**Figure 3:**
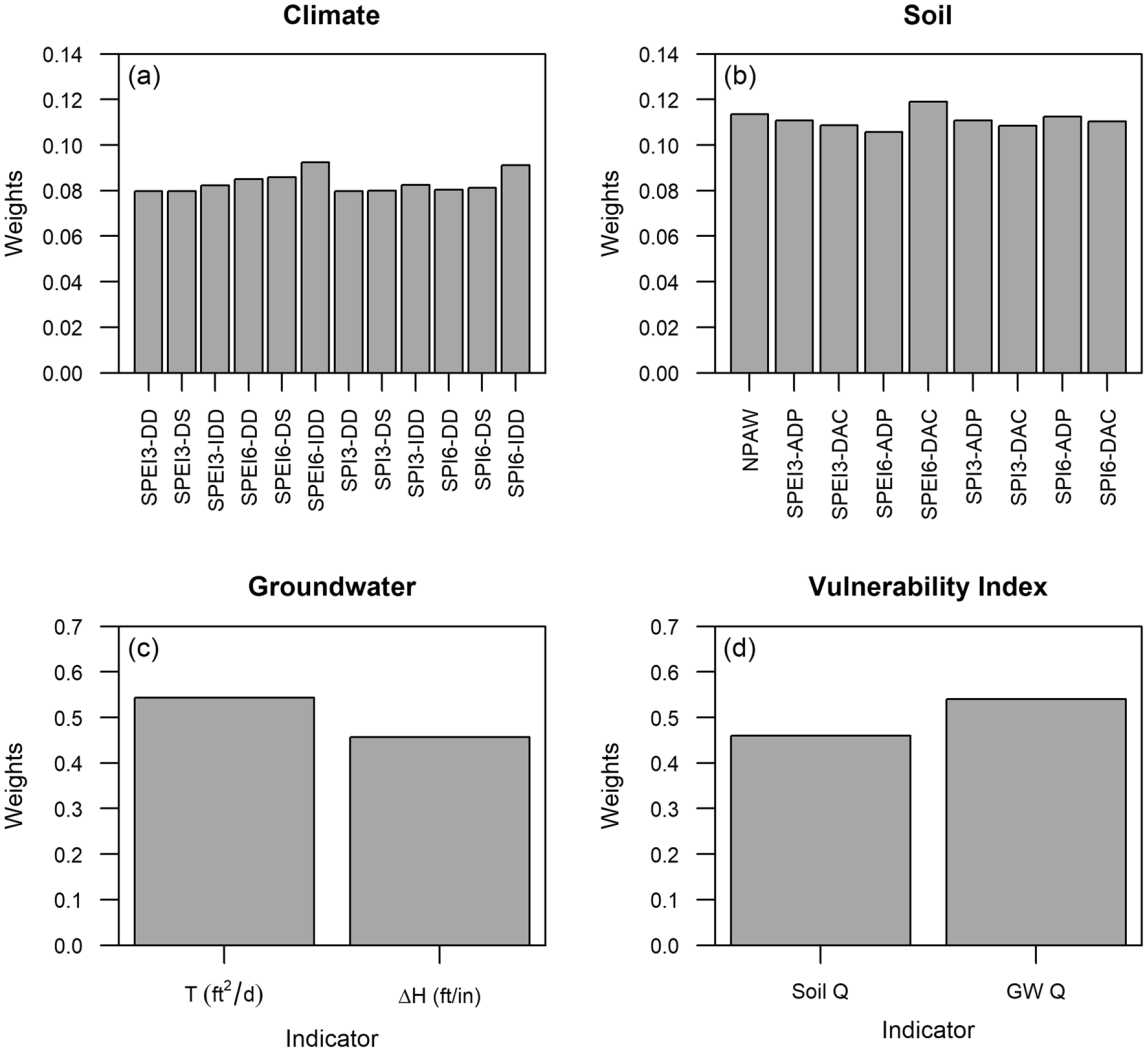
Estimated weights for various attributes used for computing (**a**) Climate Stress Index; (**b**) Soil Buffer Index; (**c**) Groundwater Buffer index and (**d**) Overall Vulnerability Index.

Map algebra routines were used to create requisite maps and intersection operations were used to extract data at the 187 grid locations depicted in Fig. 2a using ArcGIS (ESRI Inc., Redlands, CA). All data were compiled into a database for further analysis. Customized scripts were developed in R Statistical and Programming environment^71^ to compute weights using the entropy method and the final set of weights are depicted in Fig. 3.

The MCDM package^72^ was used to perform weighted multiplicative MCDM separately on climate, soil and groundwater data and the results from these individual MCDM applications were aggregated to obtain the final vulnerability index as per Eq. (1).

## Results and discussion

### Drought stresses

**Figure 4:**
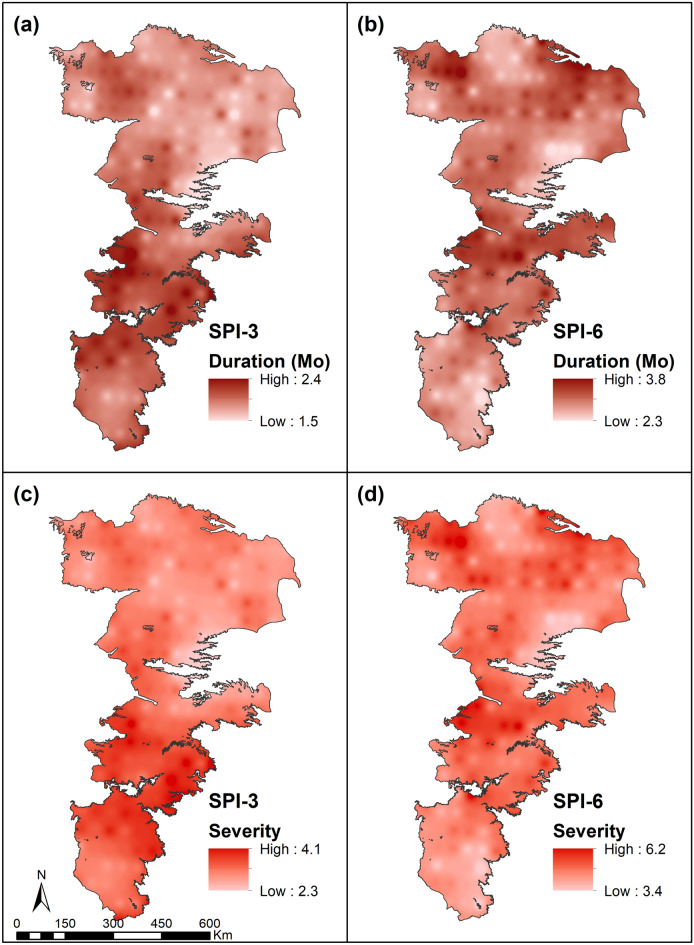
Expected values of absolute drought severity and drought duration for SPI at 3-month and 6-month accumulations.

**Figure 5:**
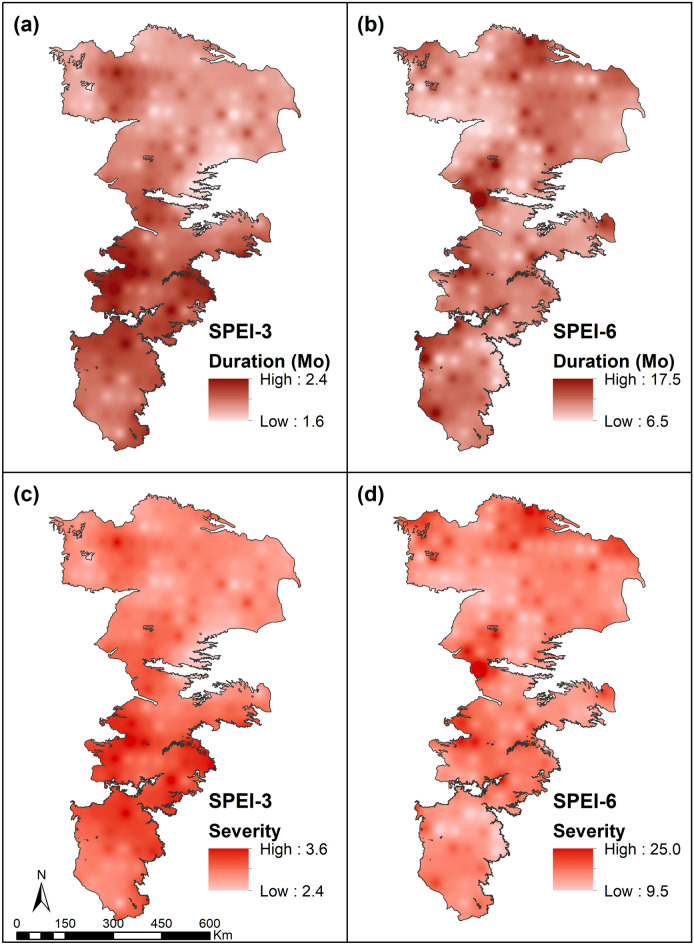
Expected values of the absolute drought severity and drought duration for SPEI at 3-month and 6-month accumulations.

A representative set of SPI and SPEI drought indicators at six different locations depicted in Fig. 2b can be found in Supplementary Information (Figs. S1 and S2). The expected values of drought duration (DD) and severity (DS) for SPI and SPEI are shown in Figs. 4 and 5. Droughts were generally temperature-controlled in the southern portions of the study area and precipitation-controlled in the northern portions. Deficit in precipitation was the primary factor affecting short-term droughts and the differences between the two indicators (SPI-3 and SPEI-3) were not significant. At 6-month accumulation, SPI droughts showed greater fluctuations and occurred sooner than those predicted by SPEI. On the other hand, SPEI droughts were prolonged highlighting the important role of PET (radiation and wind effects) in defining meteorological droughts^73^. Therefore, the combined use of these indicators allowed to capture both early initiation and prolonged persistence of meteorological droughts. The results presented in Figs. 4 and 5 indicate that short-term (3-month) droughts could extend over a significant portion of the growing season. Similarly, the long-term (6-month) droughts of both SPI and SPEI could on average extend well over the entire growing season. Long-term droughts, while not initiated as often, tend to be highly persistent in the study area.

**Figure 6:**
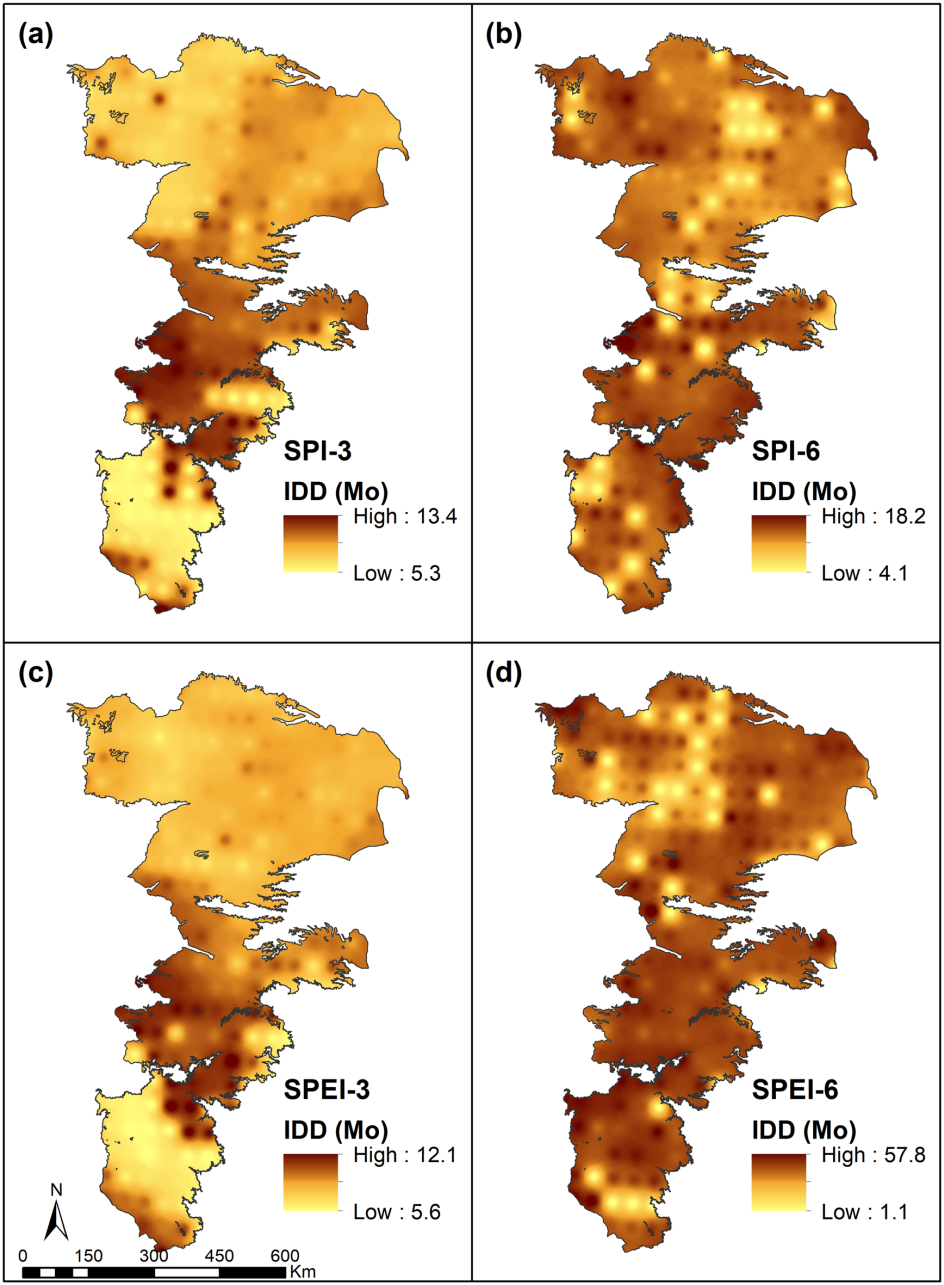
Expected value of inter-drought duration (IDD) of SPI and SPEI at 3- and 6-month accumulations.

The expected values of Inter-drought duration (IDD), shown in Fig. 6, are generally lower in the northern portions and somewhat higher in the southern portions for the 3-month accumulation. The differences between SPI and SPEI for 3-month accumulations was low as droughts at this time scale are largely precipitation controlled. The IDD for SPI-3 and SPEI-3 are higher in the central portions of the study where the drought duration is also relatively higher. These regions tend to have more stable climate states while the northern and southern portions exhibit greater transitions between drought and non-drought states. On the other hand, the variability of IDD for long-term (6-month) droughts is very high and exhibits high spatial variability. No discernable spatial trends can be noted for long-term droughts. The IDD for SPI-6 is generally shorter indicating greater variability of precipitation induced droughts, while SPEI-6 exhibits more stable climate patterns with prolonged periods of both drought and non-drought states indicating that precipitation is not strongly correlated to temperature and warmer than normal temperatures may persist over a longer period even when the region experiences some amount of precipitation.

### Soil buffering characteristics

**Figure 7:**
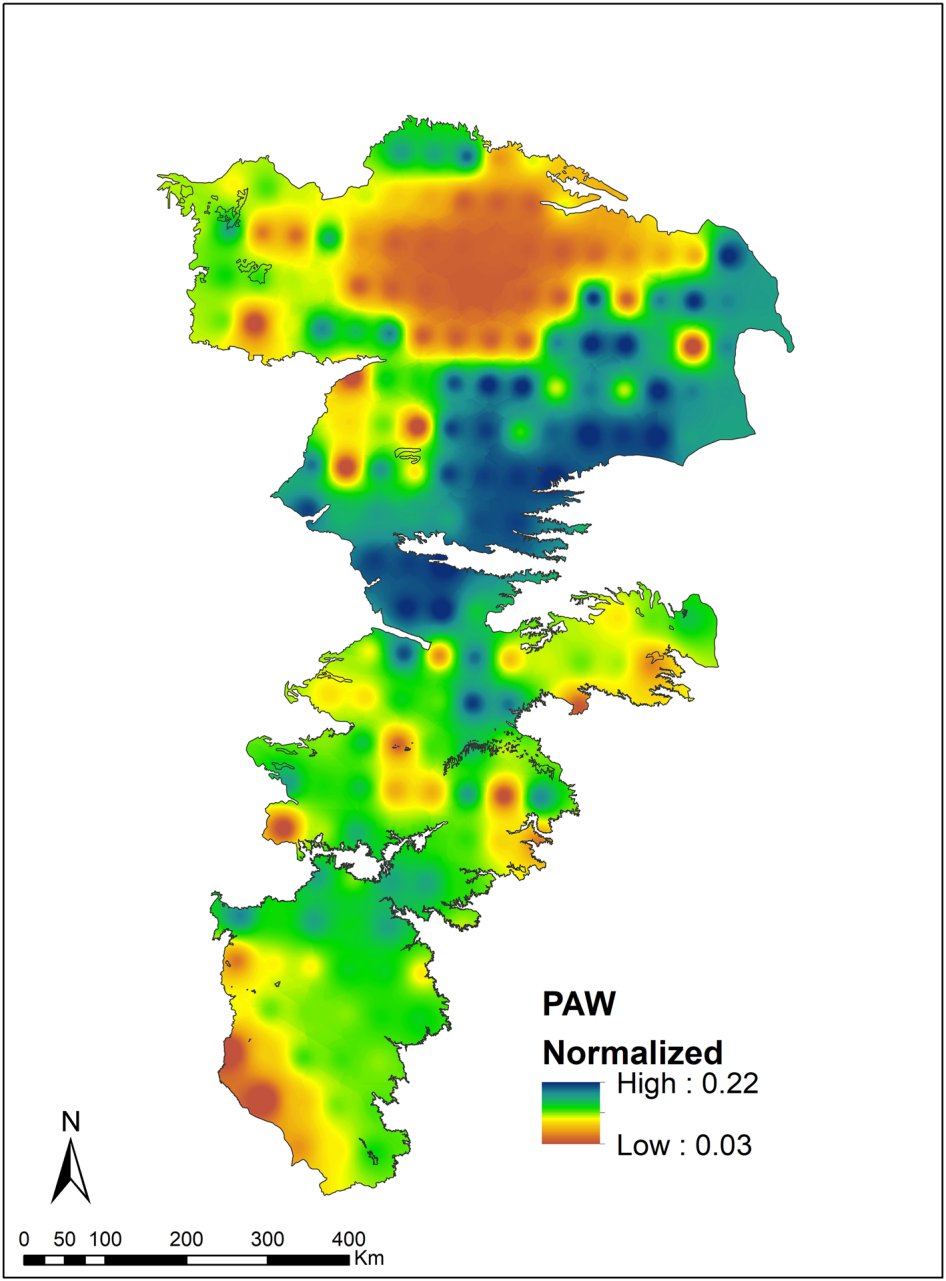
Normalized plant available water in the root zone.

The variability of Normalized Plant Available Water (NPAW) is shown in Fig. 7. The NPAW denotes the potential of the soil to hold moisture in the root zone. The soil buffering capacity is lower in the northern portions (Sandhill region of Nebraska) and in the extreme southern portions. Both these areas have sandy soils with high drainage potential. While the PAW values are low, these areas are not being used for agriculture (see Fig. 2c,d). Soil water retention is generally high in most parts of the study area with the northeast portions exhibiting the best retention characteristics.

Illustrative examples of SSMI computed for six different stations in various climate zones (see Fig. 2b for the locations of these stations) within the study area can be found in Supplementary Information (Figs. S3 and S4) along with the expected values of agricultural drought duration, severity and inter-drought duration (Fig. S5). A comparison of SSMI time-series and expected values of drought characteristics with those of meteorological droughts indicate that the initiation of agriculture droughts lag those captured by SPI and tend to be more similar to SPEI at higher accumulation periods.

**Figure 8:**
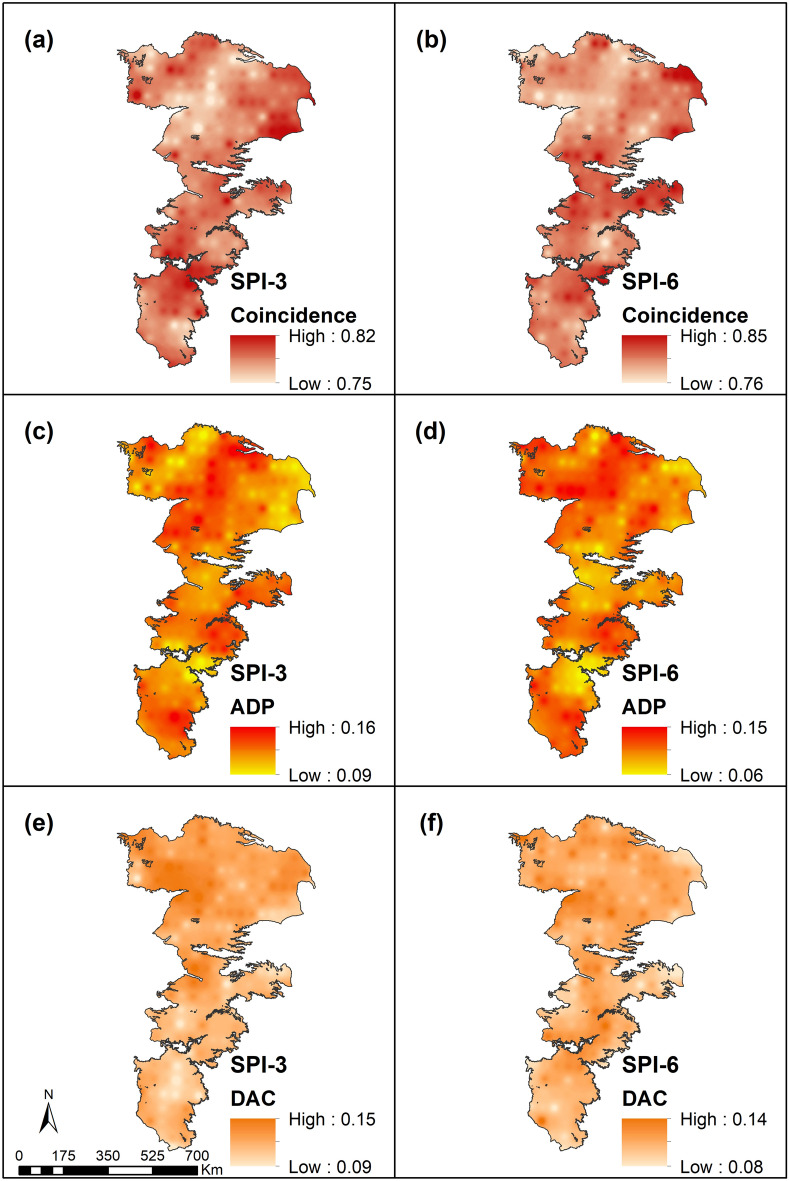
Comparison of meteorological (SPI) and agricultural (SSMI) drought indicators.

**Figure 9:**
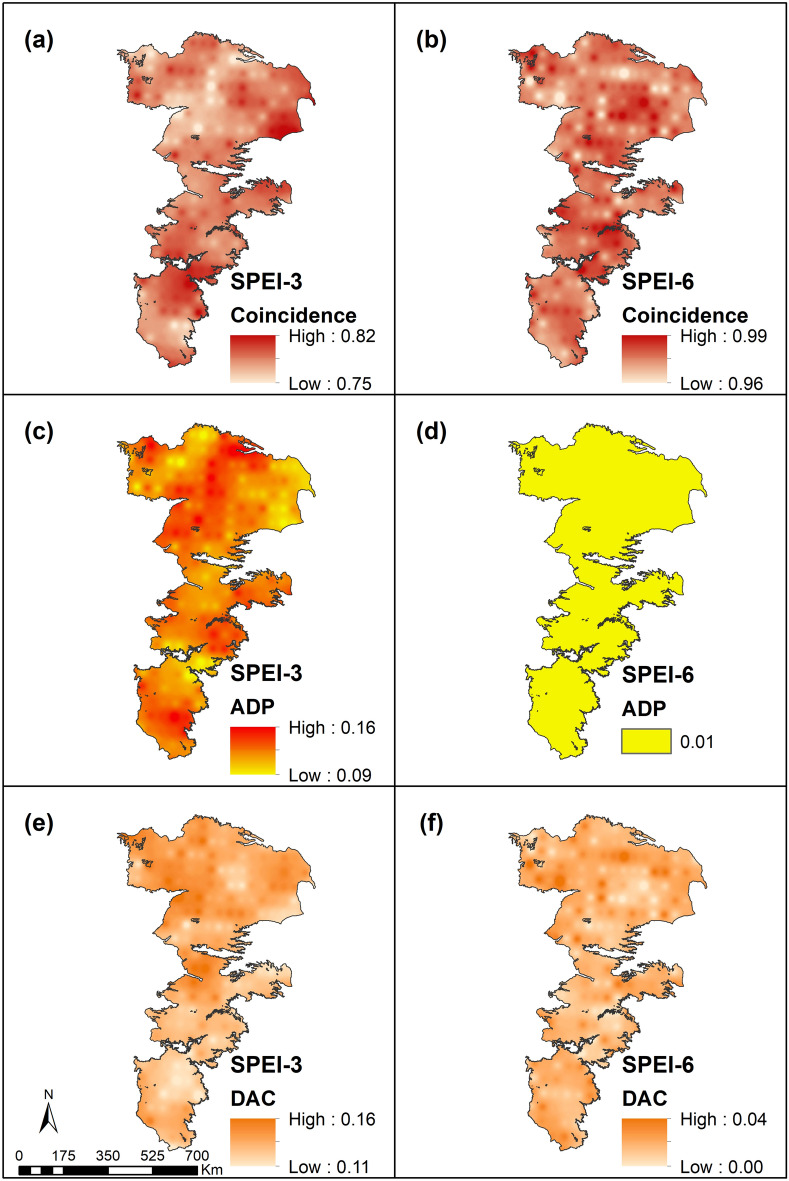
Comparison of meteorological (SPEI) and agricultural (SSMI) drought indicators.

The DAC and ADP values across the aquifer for SPI and SPEI are depicted in Figs. 8 and 9. Coincidence measures the fraction of time an APS experiences both meteorological and agricultural droughts. The coincidence between meteorological and agricultural droughts increase with increasing accumulation periods. In particular, SPEI-6 has a very high coincidence with agricultural droughts in the region and this result is to be expected because for a prolonged drought, the moisture reserves of the soil are completely depleted offering no buffering capacity.

Regardless of the indicator, the DAC and ADP exhibit higher values and greater spatial variability for short-term droughts than longer-term droughts. This result is again to be expected as the antecedent moisture conditions have a greater impact on conditioning soil moisture dynamics at shorter timescales. The result also indicates that while some shorter time-scale droughts may be overcome by precipitation events, other rainfall events may be of higher intensities and closely spaced and not contribute much to soil moisture via infiltration.

Infiltration is also limited by the fact that most of the region is covered by soils having a relatively high percentage of fines which often have sufficient storage capacities but are harder to fill by high intensity storms. In addition, rainfall events, especially during the summer tend to be of relatively short-duration and any infiltrated water is quickly lost from the soil root zone due to evapotranspiration contributing to higher ADP values. In a similar vein, the presence of fines (or smaller size pores) help retain water that is infiltrated during the cooler seasons (winter and spring) when the effects of evapotranspiration are relatively low. The range of values for DAC and ADP tend to be similar over the study area but the spatial differences between the two are better evident in regions with sandy soils (northern portions of the study area) and for higher drought accumulation levels.

### Groundwater buffering characteristics

**Figure 10:**
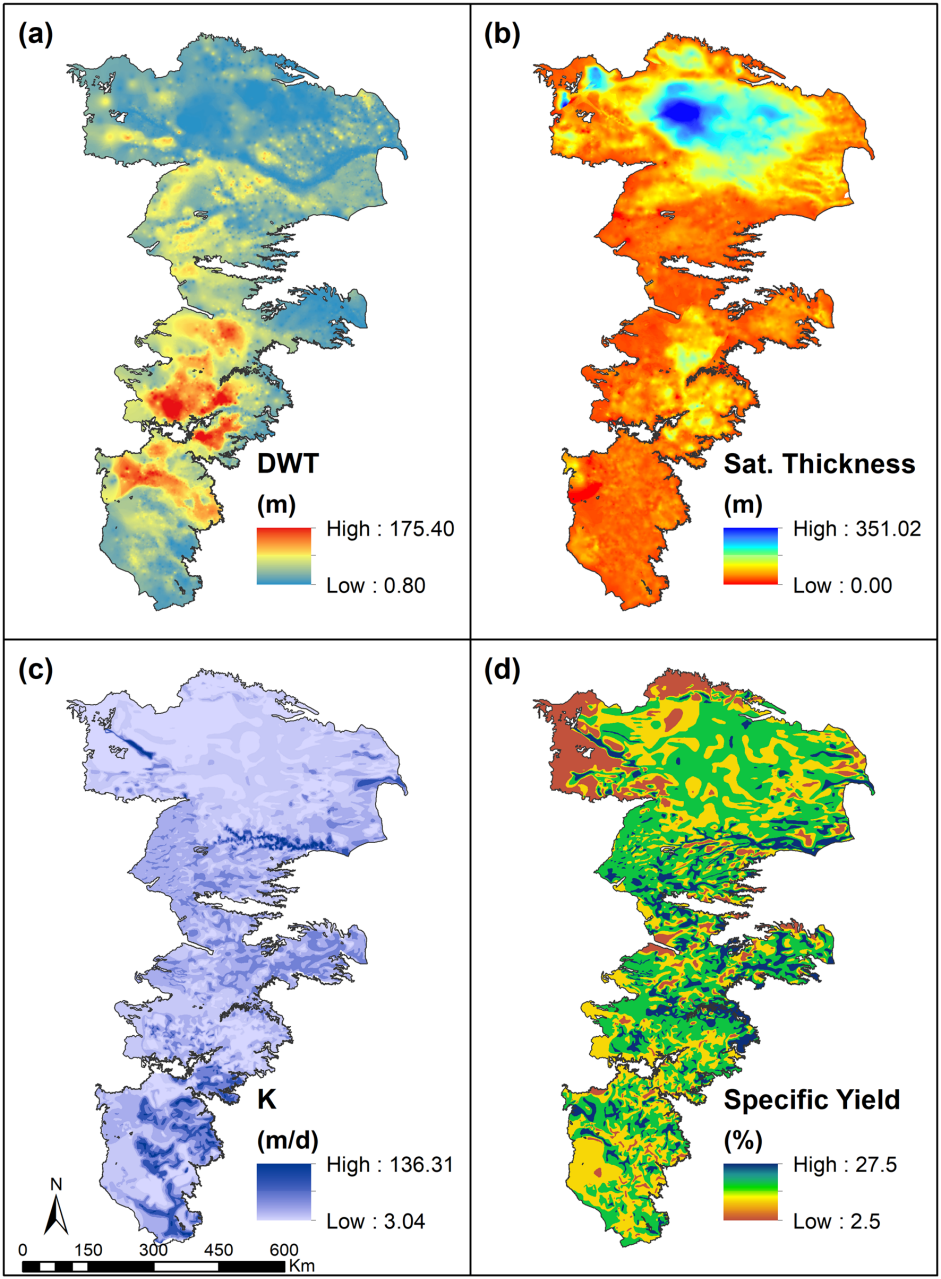
Groundwater characteristics in the study area (Data Compiled from US Geological Survey^57,68–70^).

The Groundwater characteristics of the study area are schematically depicted in Fig. 10. The depth to water table (DWT, see Fig. 10a) varies considerably across the region but exhibits the greatest depths in the central portions, especially in Texas, Oklahoma and Kansas where the groundwater has been used extensively for irrigation since the 1950s^74^. The saturated thickness of the aquifer is higher in the northern portions of the aquifer (Sandhill region of Nebraska shown by shades of blue in Fig. 10b) and the aquifer is practically depleted in the very southern portions where not only has the groundwater been over utilized but the thickness of the aquifer (intrinsic storage volume) is also relatively smaller. The hydraulic conductivity varies in an undulating manner and is locally conditioned by geological features such as the presence of paleochannels^75^ when explain the localized higher values (depicted by darker purple in Fig. 10c). The specific yield of the aquifer (shown in Fig. 10d) exhibits variability due to local scale aquifer heterogeneity but generally has a value between 0.12 and 0.18 over most of the study area.

### Vulnerability indices

**Figure 11:**
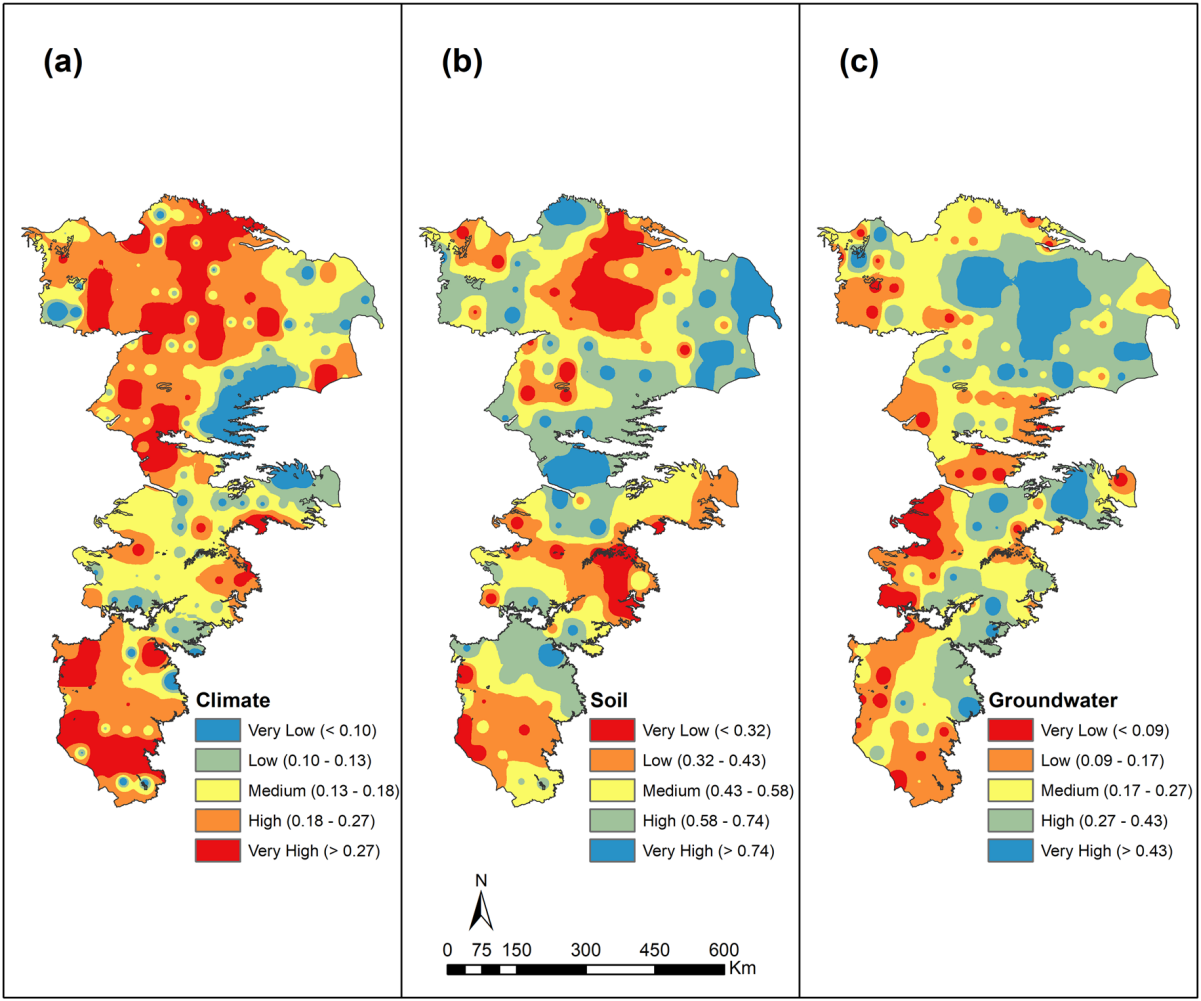
Drought Stress Index (DSI), Soil and Groundwater Buffering Indices (SBI and GBI) across the study area.

The relative climate stresses as well as soil and groundwater buffering capacities are depicted in Fig. 11. The relative indices that were normalized on a 0–1 scale were further divided into 5 categories using 20th, 40th, 60th and 80th percentiles of the computed values at the 187 grid points. The use of percentiles as thresholds allows for a consistent comparison^76^ and properly depicting the relative variability of these indices across the study area.

The Drought stress index (DSI) values depicted in Fig. 11a indicate the western portions which are more arid are subject to greater stresses than the eastern portions of the study area. Furthermore, the stresses are higher in the northern portions and the southern portions of the study area. The analysis of drought indicator characteristics (Figs. 4, 5 and 6) indicates that the stresses in the south are temperature-controlled while those in the north are precipitation-controlled.

The soil buffering capacity (Fig. 11b) exhibits a banded structure and there is relatively low buffering capacity in portions of southern, middle and northern portions. The low buffering capacity correlates well with the presence of sand content in the soils. Higher sand contents not only reduce the moisture holding capacity but also lose water via a deep percolation process. While this percolation is useful to recharge the underlying aquifers, they do not directly add to the short-term buffering capacity of the soils. The increased sand content also affects rooting depths of many plants. Such soils are generally conducive to the production of peanuts if other conditions necessary for crop growth are satisfied.

The groundwater buffer index (GBI) also exhibits a general north–south and west–east gradients across the study area (see Fig. 11c). Areas with relatively higher saturated thicknesses (Fig. 10b) are noted to exhibit higher buffering capacity but GBI is also conditioned by the depth to water table (Fig. 10a) an indicator of groundwater extraction. GBI also correlates with the temperature gradients across the region (see, Fig. 2b) with greater vulnerability in the western arid regions compared to the eastern sub-humid regions.

A net assessment of the soil and groundwater buffering capacities can be obtained by visually comparing buffering indices presented in Fig. 11b,c and contingency tables presented in Supplementary Information (Table S2). Four different spatial patterns can be seen in these comparisons: (1) The buffering capacities for both soil and groundwater are very low or low (~ 9% of the study area) as can be seen predominantly in the southern portions of the aquifer; (2) The buffering capacities of both soil and groundwater are relatively high (high or very high), as seen in the northeastern portions of the aquifer and accounts for ~ 15% of the study area; (3) Groundwater buffering capacity is relatively high (high or very high) but not those of the soils (low or very low) in some northern portions and cover ~ 13.4% of the total area; (4) Soil buffering capacity is high or very high but that of the groundwater is relatively low (~ 10% of total area). In other areas there is no single dominant buffering factor. Clearly, the worst case is when both the soil and groundwater buffering is low. As discussed earlier, the root zone has a much smaller storage capacity, so areas with higher groundwater buffering capacities but with lower soil capacities may require greater amounts of irrigation but are able to supply water more continuously to the crop compared to the situation where the buffering capacity of the soil is higher but that of the groundwater is low.

**Figure 12:**
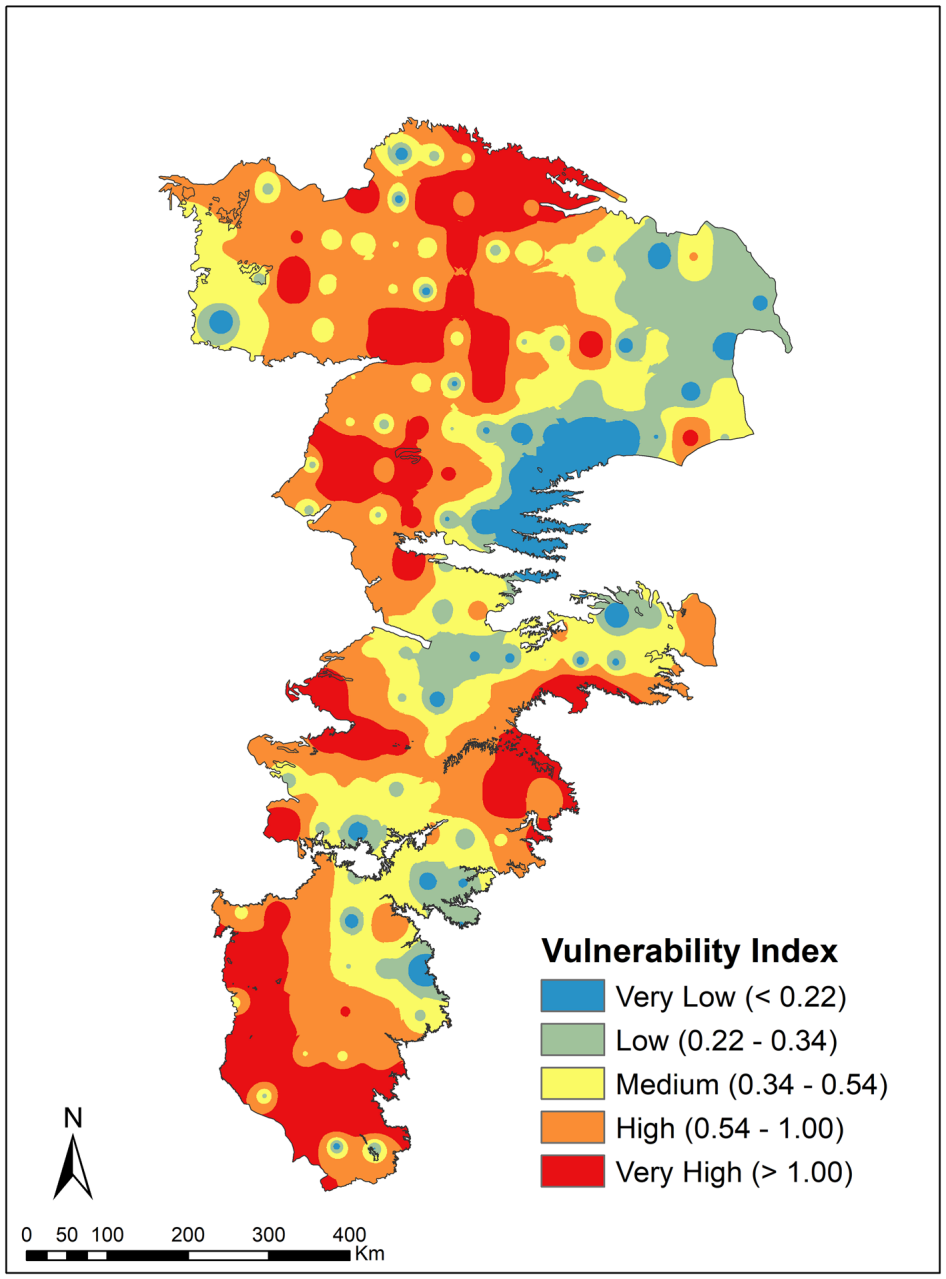
Overall Vulnerability Index to droughts for conditions in the year 2015.

The overall vulnerability index comparing the drought stresses against buffering capacity is depicted in Fig. 12. Generally, the vulnerability to droughts is higher along the western and southern of the aquifer where both climate stresses are high and buffering capacity is low. In a similar vein, the northeastern sections have lower relative vulnerability due to lower climate stresses. A little over 20% of the study area has a vulnerability index greater than 1 which suggests that the relatively buffering capacity is lower than the relative drought stress. These areas (shown in dark red in Fig. 11) are areas of highest vulnerability. A comparison of Figs. 11 and 12 indicates that the vulnerability in the northern regions are largely controlled by drought (climate) stresses while those in the southern portions are a combination of climate stresses and limited hydrologic buffering capacity. In most of these areas, irrigation may no longer be possible, and the systems have either already transitioned to, or are rapidly transitioning towards dryland farming or other land use types.

Approximately 40% of the area exhibits medium to high vulnerability where the factor of safety provided by soil and groundwater sources is less than 2 but greater than 1 (VI is > 0.5 but < 0.1). While irrigated agriculture may currently be feasible in these areas, it may not be sustainable in the long run. Deficit irrigation practices and adaptation to drought tolerant varieties will help prolong the useful life of the aquifer in these regions. In a similar token, the need for water conservation practices is the highest in these regions. Judicious crop choices and rotation of high-water use intensity (high economic value) crops with low water use intensity (low economic value) crops may be another important strategy to pursue in these areas.

Sustaining agricultural activities in the Ogallala Aquifer region is important at least in the short-term as this is the primary economic driver, which unfortunately has not resulted in other significant economic spillover effects within these rural economies^77^. Previous econometric studies that have also sought to evaluate the economic impact of droughts and groundwater availability show the importance of groundwater in areas with low rainfall and better soils^78^. The present study helps provide a refined context to such empirical studies by characterizing the competing effects of droughts and buffering provided by soils and groundwater and also help guide future econometric analysis of water use and conservation practices aimed at combating droughts.

Approximately 20% of the study area is categorized as having very low or low vulnerability. The areas with lower vulnerability are largely clustered in the northeastern section of the aquifer. These parcels benefit by relatively lower number of drought events as well as having relatively higher levels of hydrologic buffering. As such, they are prime locations for growing crops that have high economic value but also need higher amounts of water. Relatively isolated pockets of low and very low vulnerability are scattered across the study area. These pockets arise from localized heterogeneities in soils, microclimatic conditions and low anthropogenic groundwater use (e.g., ranching) in the past.

Heterogeneities in vulnerabilities of APS within a region often lead to uncertain information and affect appropriate and timely drought responses^79^. The VI map such as the one presented in Fig. 12 is helpful for farmers to understand that vulnerabilities to droughts exhibit heterogeneities and need to be tackled on a farm by farm basis. From a regional-scale water planning point of view, ‘one size fits all’ type drought contingency policies may create uneven impacts on farming communities. The developed methodology and its eventual outcome (i.e., the vulnerability map) can therefore be used to construct site-specific guidelines that balance the regional need of water curtailment during droughts vis-à-vis the local needs of meeting crop water demands.

Another very important use of the vulnerability map (Fig. 12) is evaluating the compatibility of current cropping choices against the vulnerability to droughts. The risks of crop failure (in the short run) and the eventual loss of buffering capacity due to aquifer depletion (in the long-run) arise when high water intensity crops continue to be grown in areas with high vulnerability. On the other hand, growing low water intensive crops in areas where the vulnerability is low may help sustain the aquifer in the long run but it may come at the cost of not realizing the full potential of available buffering capacity in the short-term. The locations of 5 major crops (Fig. 2d) were intersected using ArcGIS (ESRI Inc., Redlands, CA) with the vulnerability map (Fig. 12) to group cropping areas across vulnerability classes and are summarized in Table 1. Overall, the agricultural production in the region appears to be reasonably well adapted to drought vulnerability. There is a higher propensity to grow drought tolerant crops (cotton 48%, sorghum 18% and winter wheat 18%) over high water intensive crops (corn 12% and soybean 4%) in areas categorized as very highly vulnerable (VI > 1). Therefore, nearly 85% of the area categorized as very highly vulnerability have crops that require relatively lower amounts of water. Similarly, areas categorized as very low or low vulnerability generally see higher acreages of soybean (32%) and cotton (25%). However, the potential to further harmonize cropping choices with climate risks and hydrologic buffering capacities exist across the aquifer. The areas categorized as medium to high vulnerability are regions that exhibit the highest potential to increased vulnerability in the future. The crop area categorized as exhibiting medium to high vulnerability have a mix of 25% high water intensive crops (corn 16% and soybean 9%) and 75% moderate to low water intensive crops (cotton 27%, sorghum 22%, winter wheat 26%) indicating that adaption is also occurring in these areas. The viability of sustaining corn and soybean production in these areas will depend upon adoption of conservation strategies (irrigation scheduling and high efficiency water application methods) along with better cultivars that exhibit greater resilience to water stresses.

**Table 1:** Percentage of crop production area under each vulnerability class for five major crops grown in the ogallala aquifer region.

Vulnerability classification	Percent of crop production area under each vulnerability class				
	Corn (%)	Cotton (%)	Sorghum (%)	Soybean (%)	Winter Wheat (%)
Very low	14.82	1.71	10.00	19.47	7.07
Low	47.88	18.79	39.25	62.05	34.59
Medium	18.56	23.88	26.60	12.60	30.63
High	9.87	22.03	11.35	3.23	15.08
Very high	8.87	33.58	12.79	2.65	12.63
Total area (sq. Km)	59,355.77	19,246.66	12,135.91	14,665.33	39,468.53

## Summary and conclusions

A new vulnerability index (VI) is defined in this study using the concept of robustness. The proposed VI compares the stresses caused by droughts against the robustness (buffering) provided by soil and groundwater sources using a multicriteria decision making (MCDM) framework. Physical principles are used to define drought stress index (DSI) as well as soil buffer index (SBI) and groundwater buffer index (GBI). In addition, an entropy-based scheme is utilized to objectively weigh the relative contributions of parameters used to define stresses and buffers. Furthermore, DSI, SBI and GBI indices can be computed using easily available data and integrated within a geographic information system (GIS) to map relative vulnerabilities of land parcels within a region to droughts.

The developed approach is used to map current (year 2015 baseline) drought vulnerabilities in the area underlain by the Ogallala Aquifer, which is the largest aquifer in the US. The results of the study indicate that the vulnerabilities in the north are caused by precipitation related moisture deficits and limited moisture holding capacity of soils. While, vulnerabilities in the southern portions are brought forth by temperature driven moisture deficits and limited groundwater availability. While current crop growth choices are compatible with the intrinsic drought vulnerabilities nearly 50% of the aquifer region falls in the transition zone between low and very high vulnerabilities. Better management of groundwater in these areas is critical to sustain APS in the long-run.

Adaptation of APS to droughts is important as the population of the world continues to rise and changes in climate are likely to result in high intensity and more prolonged droughts. Understanding the robustness of the farm systems is fundamental to assess their ability to withstand droughts and foster nexus-based water management^80^ wherein cropping choices are harmonized with water availability (robustness), drought risks (stresses). The present study offers a methodology to elucidate robustness and provide insights on factors controlling the vulnerability of these systems to droughts.

## Supplementary Information

Supplementary Information.
