# Supplementary material for: Vulnerability assessment of agricultural production systems to drought stresses using robustness measures
Source: Sci Rep. 2021 Nov 4;11:21648. doi: 10.1038/s41598-021-98829-5 (PMC8568896; doi:10.1038/s41598-021-98829-5)
Supplement: Supplementary file 1 — Supplementary Information. [file 41598_2021_98829_MOESM1_ESM.docx]

## Vulnerability Assessment of Agricultural Production Systems to Drought Stresses using Robustness Measures

Marangely Gonzalez Cruz, E. Annette Hernandez*, Venkatesh Uddameri

Department of Civil, Environmental and Construction Engineering, Texas Tech University, Lubbock, TX 79409-1023

*Corresponding author: Annette.Hernandez@ttu.edu

Table S1: Datasets Used in this Study Along with their Sources

| Data | Description | Source | URL |
| --- | --- | --- | --- |
| Precipitation | Monthly precipitation on a 0.5° x 0.5° grid (NCDF), 1901-2018. | Climate Research Unit (CRU) TS V 4.03^1^ | https://crudata.uea.ac.uk/cru/data/hrg/ |
| Potential Evapotranspiration | Monthly potential evapotranspiration on a 0.5° x 0.5° grid (NCDF), 1901-2018 | Climate Research Unit (CRU) TS V 4.03^1^ | https://crudata.uea.ac.uk/cru/data/hrg/ |
| Climate Classification | World Map of Köppen-Geiger Climate Classification at 1 km resolution (raster) | Present and future Köppen-Geiger climate classification maps^2^ | https://figshare.com/articles/Present_and_future_K_ppen-Geiger_climate_classification_maps_at_1-km_resolution/6396959/2 |
| Soil Moisture | Monthly soil moisture on a 0.5° x 0.5° grid (NCDF), 1948-2019 | Climate Prediction Center (CPC) Soil Moisture V2^3^ | https://psl.noaa.gov/data/gridded/data.cpcsoil.html |
| Root Zone Depth | Average root zone depth values for commodity crops for major earthy components, cm. Raster on a 10m x 10m grid. | Gridded Soil Survey Geographic (gSSURGO) by the USDA-NRCS^4-11^ | https://gdg.sc.egov.usda.gov/GDGOrder.aspx?order=QuickState |
| Available Water Storage in the Root Zone | Average available water storage within the root zone depth for major earthy components, mm. Raster on a 10m x 10m grid. | Gridded Soil Survey Geographic (gSSURGO) by the USDA-NRCS^4-11^ | https://gdg.sc.egov.usda.gov/GDGOrder.aspx?order=QuickState |
| Water Level | Water level data in the Ogallala aquifer for the 8 states by hydrologic year from 1996 to 2013. Text file based on well locations. | USGS Nebraska Water Science Center^12^ | https://ne.water.usgs.gov/projects/HPA/data.html |
| Saturated Thickness | Digital map of saturated thickness, High Plains aquifer, 2009. Raster on a 500m x 500m grid. | U.S. Geological Survey^13^ | https://water.usgs.gov/GIS/metadata/usgswrd/XML/sir12-5177_hp_satthk09.xml#stdorder |
| Water Level Changes (1950-2015) | Spatial data set of mapped water-level changes in the High Plains aquifer, predevelopment (about 1950) to 2015. Raster on a 500m x 500m grid. | U.S. Geological Survey^12^ | https://www.sciencebase.gov/catalog/item/58ff75a2e4b006455f2d6327 |
| Water Level Changes (2013-2015) | Spatial data set of mapped water-level changes in the High Plains aquifer, 2013 to 2015. Raster on a 500m x 500m grid. | U.S. Geological Survey^12^ | https://www.sciencebase.gov/catalog/item/58ff757ee4b006455f2d6325 |
| Hydraulic Conductivity | Digital map of hydraulic conductivity for the High Plains Aquifer. Vector data containing upper and lower values in ft/d. | U.S. Geological Survey^14^ | https://water.usgs.gov/GIS/metadata/usgswrd/XML/ofr98-548.xml#stdorder |
| Specific Yield | Digital map of specific yield ranges in the High Plains aquifer of the United States. Raster on a 500m x 500m grid. | U.S. Geological Survey^15^ | https://water.usgs.gov/GIS/metadata/styles/landingPage/sir12-5177_hp_sp_yield.xml#stdorder |
| Land Surface Datum | Digital elevation models (DEMs) at approx. 10m resolution (1/3 arc-second DEM) | USGS. 3D Elevation Program (3DEP)^16^ | https://viewer.nationalmap.gov/basic/ |
| Land Use/Land Cover | National Land Cover Database (NLCD), nationwide data on land cover at a 30m resolution, CONUS 2016. Raster on a 30m x 30m grid. | Multi-Resolution Land Characteristics  Consortium (MRLC)^17,18^ | https://www.mrlc.gov/data |
| Cropland | Cropland Data Layer (CDL), agricultural land cover over the Continental United States at 30 meters resolution for 2019. Raster on a 30m x 30m grid. | USDA National Agricultural Statistics Service (NASS)^19^ | https://www.nass.usda.gov/Research_and_Science/Cropland/Release/index.php |

Table S2: Contingency Table of the Soil and Groundwater Buffering Indices

|  | | **GBI** | | | | |
| --- | --- | --- | --- | --- | --- | --- |
|  |  | **Very Low** | **Low** | **Medium** | **High** | **Very High** |
| **SBI** | **Very Low** | 6862 | 32410 | 49473 | 51162 | 47777 |
|  | **Low** | 43143 | 81152 | 151035 | 91955 | 53093 |
|  | **Medium** | 25463 | 128308 | 144968 | 146410 | 78821 |
|  | **High** | 13422 | 119908 | 170632 | 180286 | 34887 |
|  | **Very High** | 9039 | 44111 | 62696 | 45660 | 9221 |


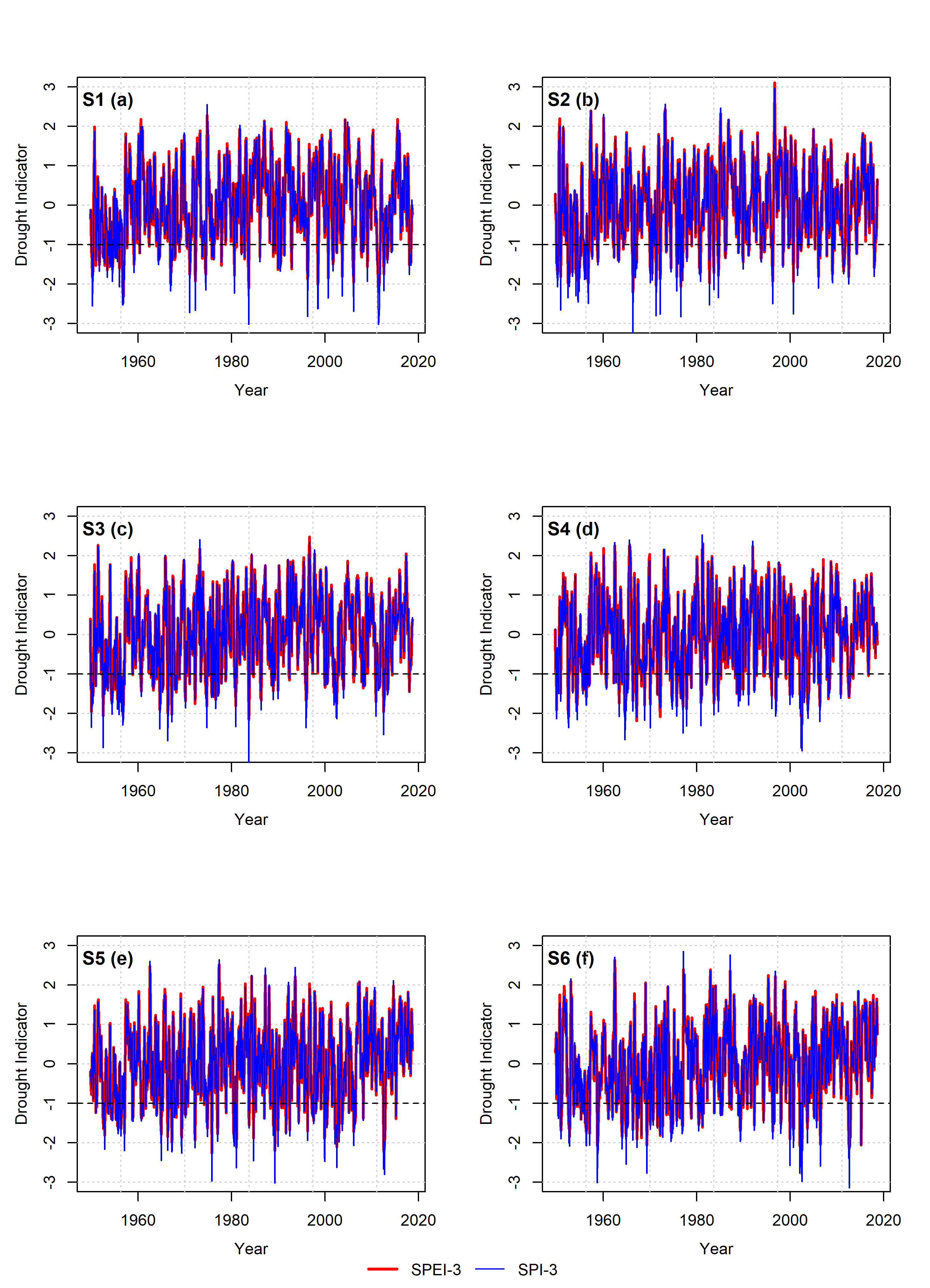


Figure S1: SPI-3 and SPEI-3 drought indicators at six different locations identified in Figure 2(b)


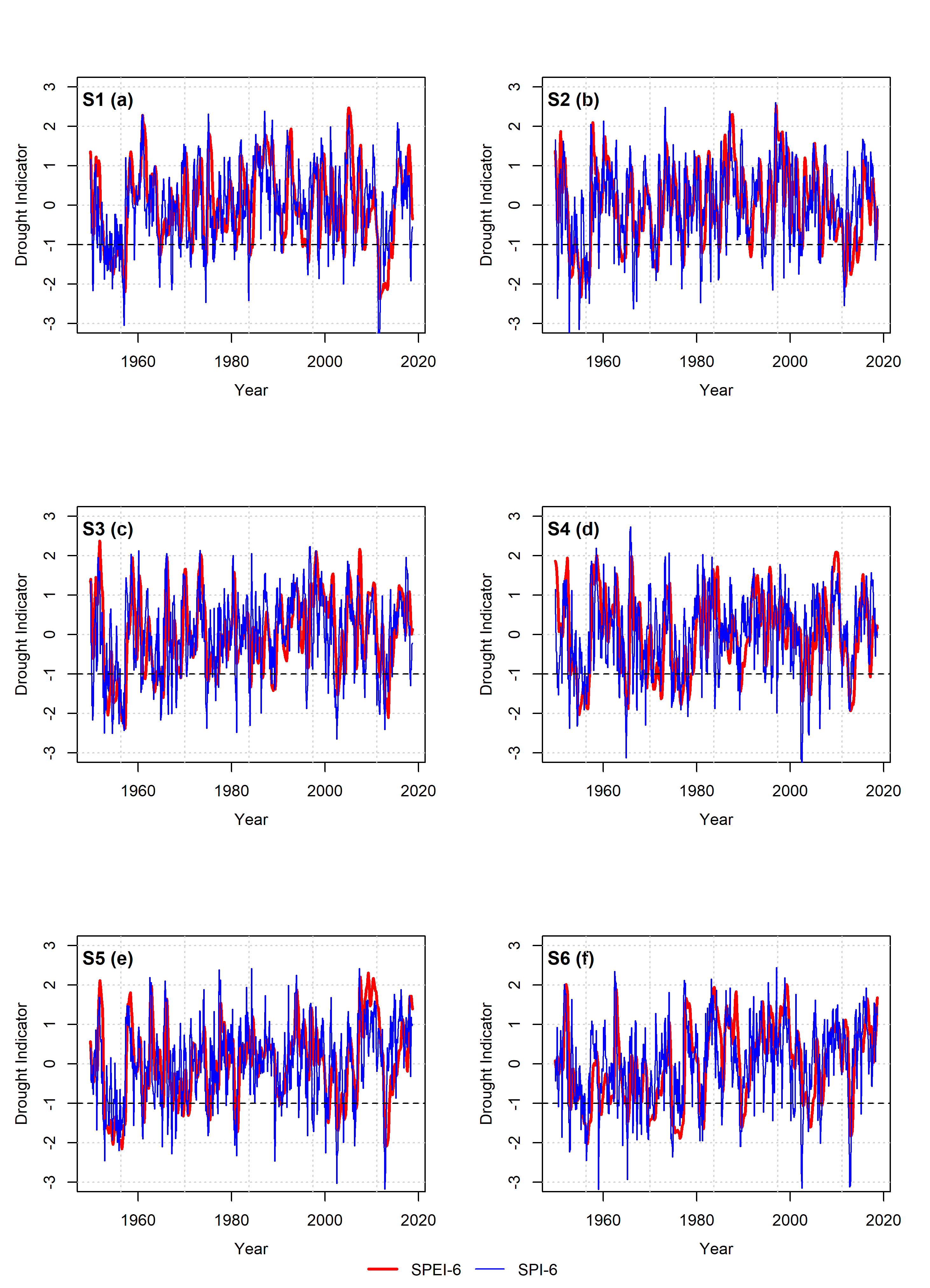


Figure S2: SPI-6 and SPEI-6 drought indicators at six different locations identified in Figure 2(b)


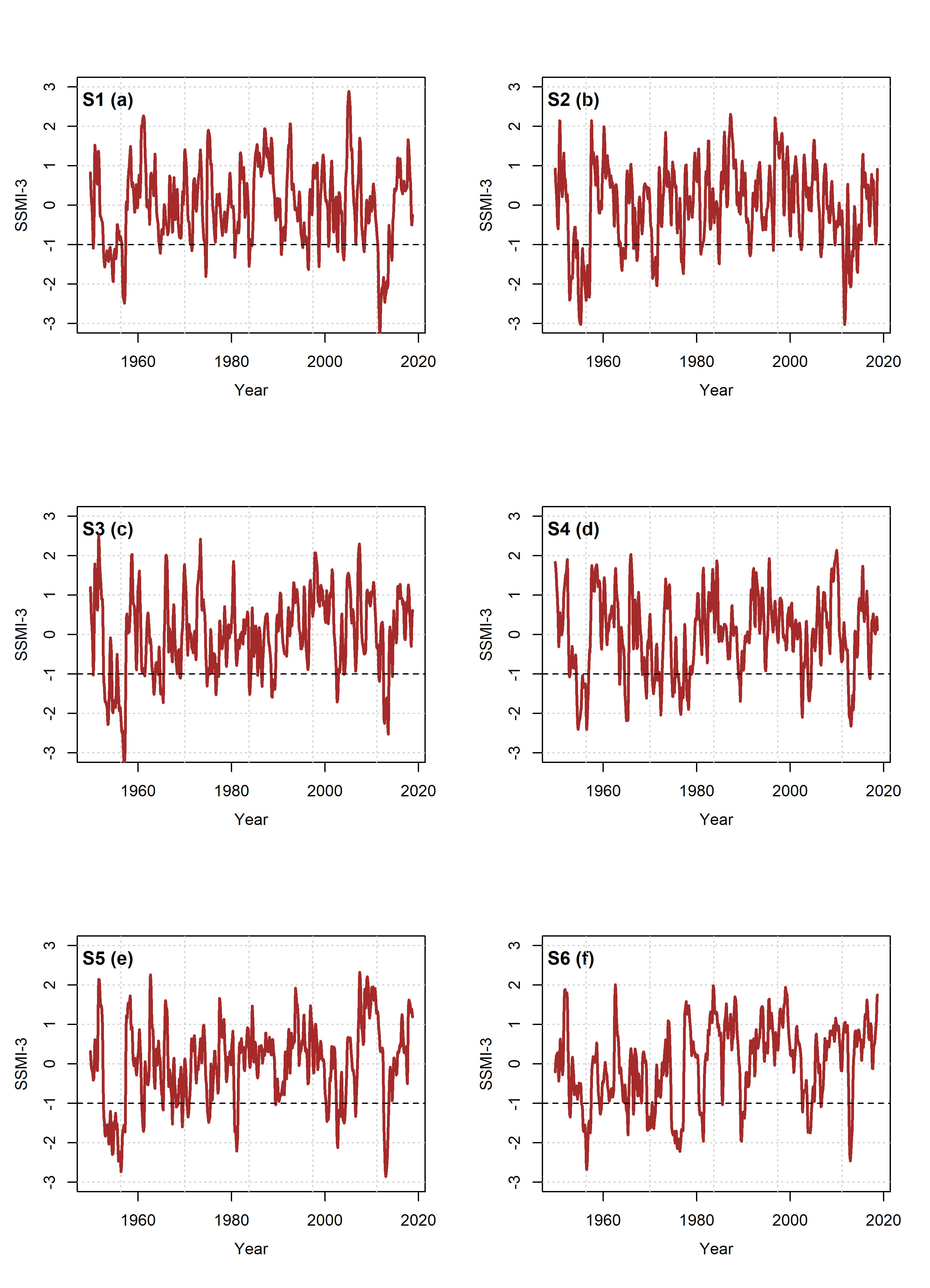


Figure S3: SSMI-3 drought indicator at six different locations identified in Figure 2(b)


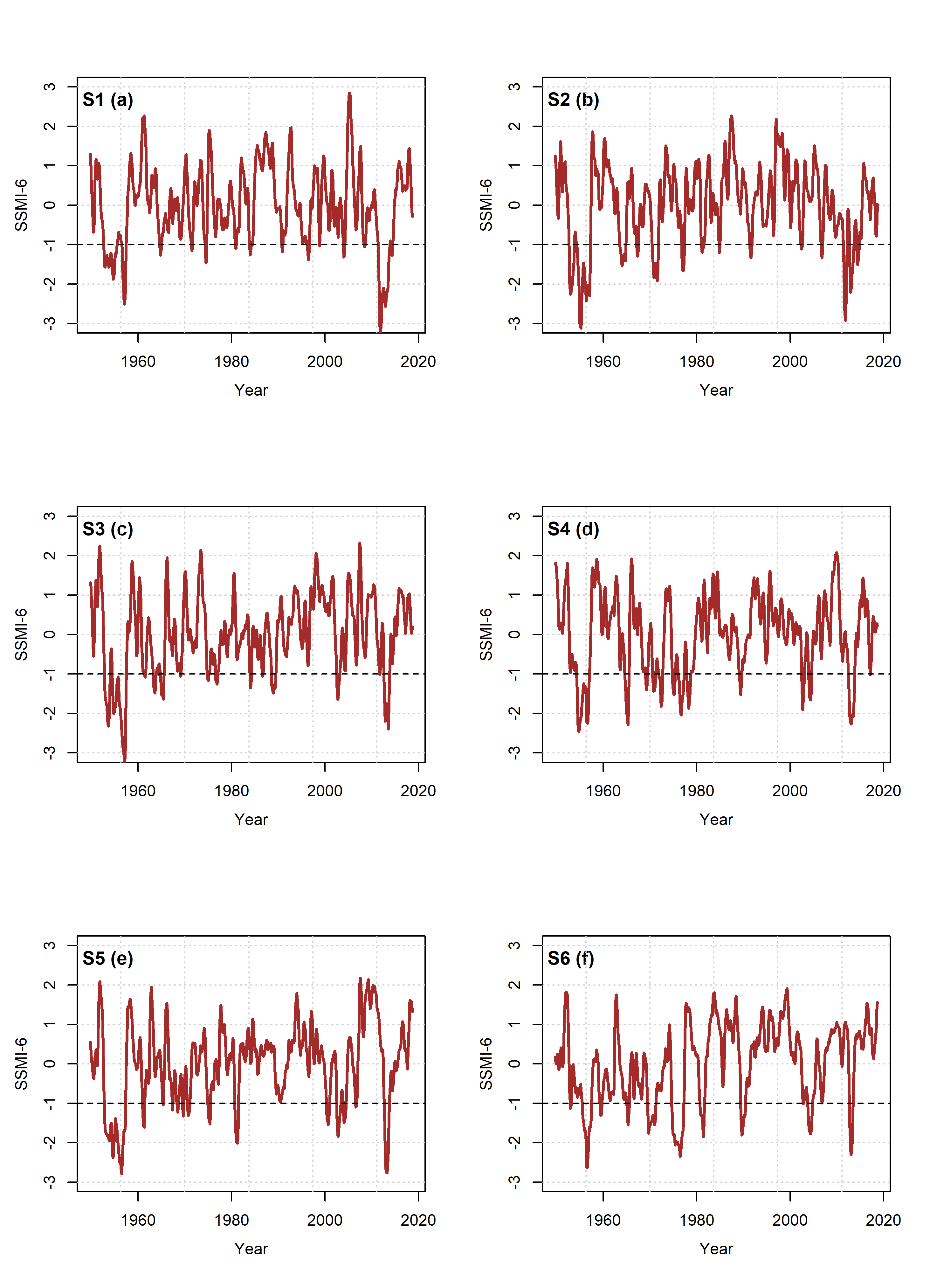


Figure S4: SSMI-6 drought indicator at six different locations identified in Figure 2(b)


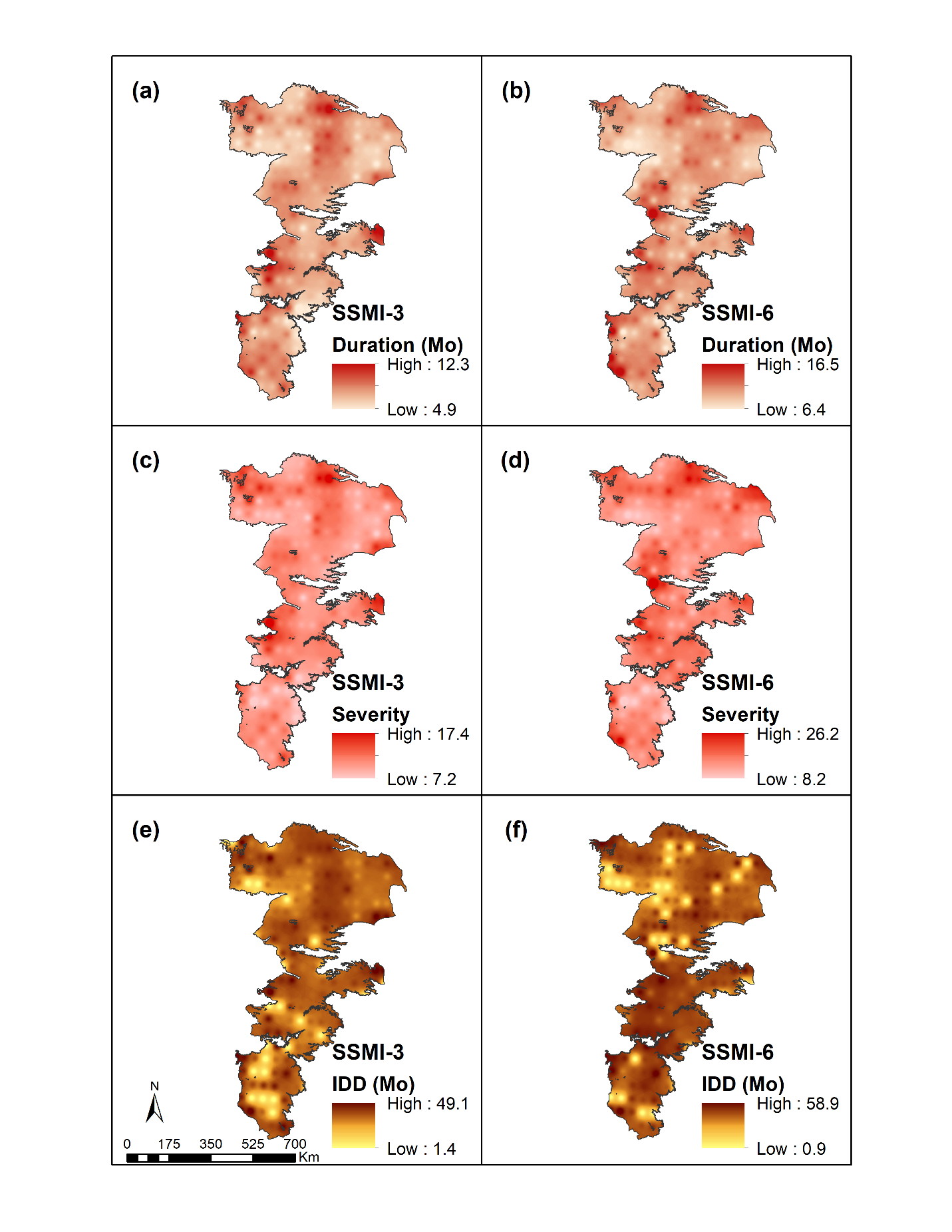


Figure S5: Expected Values of Drought Duration, Drought Severity and Inter Drought Duration for SSMI at 3-month and 6-month Accumulations

## References

1 Harris, I., Osborn, T. J., Jones, P. & Lister, D. Version 4 of the CRU TS monthly high-resolution gridded multivariate climate dataset. *Scientific data* **7**, 1-18 (2020).

2 Beck, H. E. *et al.* Present and future Köppen-Geiger climate classification maps at 1-km resolution. *Scientific data* **5**, 180214 (2018).

3 Fan, Y. & Van Den Dool, H. Climate Prediction Center global monthly soil moisture data set at 0.5 resolution for 1948 to present. *Journal of Geophysical Research: Atmospheres* **109** (2004).

4 Soil Survey Staff. *Gridded Soil Survey Geographic (gSSURGO) Database for Colorado. United States Department of Agriculture, Natural Resources Conservation Service.* Available online at <https://gdg.sc.egov.usda.gov/>. Accessed 29 Apr. 2020. (2019).

5 Soil Survey Staff. *Gridded Soil Survey Geographic (gSSURGO) Database for Kansas. United States Department of Agriculture, Natural Resources Conservation Service.* Available online at <https://gdg.sc.egov.usda.gov/>. Accessed 29 Apr. 2020. (2019).

6 Soil Survey Staff. *Gridded Soil Survey Geographic (gSSURGO) Database for Nebraska. United States Department of Agriculture, Natural Resources Conservation Service.* Available online at <https://gdg.sc.egov.usda.gov/>. Accessed 29 Apr. 2020. (2019).

7 Soil Survey Staff. *Gridded Soil Survey Geographic (gSSURGO) Database for New Mexico. United States Department of Agriculture, Natural Resources Conservation Service.* Available online at <https://gdg.sc.egov.usda.gov/>. Accessed 29 Apr. 2020. (2019).

8 Soil Survey Staff. *Gridded Soil Survey Geographic (gSSURGO) Database for Oklahoma. United States Department of Agriculture, Natural Resources Conservation Service.* Available online at <https://gdg.sc.egov.usda.gov/>. Accessed 29 Apr. 2020. (2019).

9 Soil Survey Staff. *Gridded Soil Survey Geographic (gSSURGO) Database for South Dakota. United States Department of Agriculture, Natural Resources Conservation Service.* Available online at <https://gdg.sc.egov.usda.gov/>. Accessed 29 Apr. 2020. (2019).

10 Soil Survey Staff. *Gridded Soil Survey Geographic (gSSURGO) Database for Texas. United States Department of Agriculture, Natural Resources Conservation Service.* Available online at <https://gdg.sc.egov.usda.gov/>. Accessed 29 Apr. 2020. (2019).

11 Soil Survey Staff. *Gridded Soil Survey Geographic (gSSURGO) Database for Wyoming. United States Department of Agriculture, Natural Resources Conservation Service.* Available online at <https://gdg.sc.egov.usda.gov/>. Accessed 29 Apr. 2020. (2019).

12 McGuire, V. L. Water-level and recoverable water in storage changes, high plains aquifer, predevelopment to 2015 and 2013–15. Report No. 2328-0328, (US Geological Survey, 2017).

13 McGuire, V., Lund, K. & Densmore, B. Saturated Thickness, High Plains Aquifer, 2009. *US Geological Survey Scientific Investigations Report issue ID: USGS SIR 2012‐5177* (2012).

14 Cederstrand, J. R. & Becker, M. F. Digital map of hydraulic conductivity for the High Plains Aquifer in parts of Colorado, Kansas, Nebraska, New Mexico, Oklahoma, South Dakota, Texas, and Wyoming. Report No. 2331-1258, (1998).

15 McGuire, V., Lund, K. & Densmore, B. Specific yield, High Plains Aquifer. *USGS Scientific Investigations Report* **5177** (2012).

16 Sugarbaker, L. J. *et al.* Status of the 3D Elevation Program, 2015. Report No. 2331-1258, (US Geological Survey, 2017).

17 Yang, L. *et al.* A new generation of the United States National Land Cover Database: Requirements, research priorities, design, and implementation strategies. *ISPRS journal of photogrammetry and remote sensing* **146**, 108-123 (2018).

18 Homer, C. *et al.* Conterminous United States land cover change patterns 2001–2016 from the 2016 National Land Cover Database. *ISPRS Journal of Photogrammetry and Remote Sensing* **162**, 184-199 (2020).

19 USDA-NASS. *2019 Cropland Data Layer.* Available at <https://nassgeodata.gmu.edu/CropScape/>. Accessed 29 Apr. 2020. (2020).
